# mPEG-PCL Nanoparticles to Improve Oral Bioavailability of Acalabrutinib: Effect of Polymer Lipophilicity and Hydrophilicity on Physicochemical Properties and In Vivo Performance in Rats

**DOI:** 10.3390/pharmaceutics17060774

**Published:** 2025-06-13

**Authors:** Swagata Sinha, Punna Rao Ravi, Sahadevan Rajesh Rashmi, Łukasz Szeleszczuk

**Affiliations:** 1 Department of Pharmacy, Birla Institute of Technology and Science, Hyderabad Campus, Jawahar Nagar, Kapra Mandal, Medchal District, Pilani 500078, Telangana, India; p20210055@hyderabad.bits-pilani.ac.in (S.S.); p20220224@hyderabad.bits-pilani.ac.in (S.R.R.); 2 Department of Organic and Physical Chemistry, Faculty of Pharmacy, Medical University of Warsaw, 1 Banacha Str., 02-093 Warsaw, Poland; lszeleszczuk@wum.edu.pl

**Keywords:** co-polymerization, amphiphilic co-polymer, central composite design, nanoparticles, oral pharmacokinetics

## Abstract

**Background/Objectives:** This research focuses on the development and optimization of polymer–lipid hybrid nanoparticles (PLHNs) using two grades of mPEG-PCL co-polymers in combination with DPPC and lecithin to address the biopharmaceutical challenges of acalabrutinib (ACP), a selective treatment for different hematological malignancies. **Methods**: Variations in the mPEG-to-ε-caprolactone ratio influenced both the molecular weight (Mw) of the synthesized co-polymers and their aqueous phase affinity. The ACP-loaded PLHNs (ACP-PLHNs) were optimized using a circumscribed central composite design. The in vivo studies were performed in Wistar rats. **Results**: The lipophilic mPEG-PCL (Mw = 9817.67 Da) resulted in PLHNs with a particle size of 155.91 nm and 40.08% drug loading, while the hydrophilic mPEG-PCL (Mw = 23,615.84 Da) yielded PLHNs with a relatively larger size (223.46 nm) and relatively higher drug loading (46.59%). The drug release profiles were polymer-grade dependent: lipophilic ACP-PLHNs (*l*ACP-PLHNs) sustained release up to 30 h in pH 7.2 buffer, while hydrophilic ACP-PLHNs (*h*ACP-PLHNs) completed release within 24 h. Stability studies showed greater stability for *l*ACP-PLHNs, likely due to reduced molecular rearrangement from the chemically stable lipophilic co-polymer. **Conclusions**: Oral administration of both formulations exhibited a 2-fold (*p* < 0.001) improvement in the C_max_ and AUC_0-tlast_ and a 3.9-fold (*p* < 0.001) increase in the relatively oral bioavailability compared to the conventional ACP suspension in male wistar rats.

## 1. Introduction

Polymer–lipid hybrid nanoparticles (PLHNs) have emerged as a prominent choice in the growing field of nanoparticle-based drug delivery systems (NDDSs), owing to the unique combination of benefits they offer. By merging the advantages of both polymers and lipids, PLHNs allow for precise control over critical factors such as drug release and polydispersity, which are facilitated by polymers, while lipids contribute biomimetic properties that enhance biocompatibility [1,2]. In contrast, NDDSs formulated from either polymers or lipids alone have notable drawbacks. Polymer-based NDDSs often suffer from the generation of toxic by-products during degradation; rapid elimination from the body due to the hydrophobic components’ foreign nature; and limited drug loading capacity [1,3]. Lipid-based NDDSs, on the other hand, tend to exhibit issues like burst drug release, instability during storage, and a broad size distribution [2,3]. PLHNs are created through a coherent physical hybridization of both a polymer(s) (such as polycaprolactone; polylactic acid; polylactic-co-glycolic acid; polymethacrylic acid; alginic acid; hyaluronic acid; etc.) and a lipid(s) (such as phospholipids, 1,2-distearoyl-glycero-3-phosphocholine, 1,2-distearoyl-glycero-3-phosphoethanolamine, etc.; cationic lipids, 1,2-dioleoyl-3-trimethylammonium propane, 1,2-dipalmitoyl-3-trimethylammonium-propane, etc.; fatty acids, lauric acid, myristic acid, oleic acid, etc.; and glycerides, glyceryl monostearate, glyceryl dibehenate, etc.) [3,4]. This hybrid system is further stabilized using a surfactant(s), such as Tween 80; polyvinyl alcohol; sodium cholate; lecithin; poloxamine; etc. Moreover, conjugation of either the polymer or lipid with various functional ligands, such as methyl ether polyethylene glycol (mPEG); folic acid; hyaluronan; etc., provides advantages such as stealthness; prolonged circulation; enhanced tumor targeting or uptake; etc. [5,6,7,8,9]. Of all these, the co-polymerization of mPEG with ε-caprolactone (resulting in mPEG-PCL block polymers) has extensively been used for the efficient delivery of various critical small or bio-based molecules [8,9,10]. The addition of mPEG, a hydrophilic moiety, to PCL enhances its amphiphilicity and, in turn, its biodegradability [9]. The co-polymer has been extensively used as a carrier system for various drugs (methotrexate; carbazitaxel; sulforaphane; and doxorubicin) indicated for different types of carcinoma, offering advantages such as high drug encapsulation and loading [11,12]; sustained release [12]; longer circulation time due to the stealth effect of mPEG [13]; and enhanced storage stability [11]. Further, the nanoparticulate systems formulated using mPEG-PCL have resulted in the improvement of solubility and bioavailability of several BCS class II/IV molecules [8,14,15].

Acalabrutinib (ACP), a US FDA-approved drug, is indicated for the treatment of both naïve and relapsed/refractory chronic lymphocytic leukemia (CLL) or small lymphocytic lymphoma [16,17]. As a first-line therapy, it offers a favorable safety profile with minimal adverse drug reactions and is marketed under the brand name Calquence (100 mg capsules and tablets containing ACP maleate salt) by AstraZeneca, Cambridge, UK. Despite its clinical advantages, ACP faces several biopharmaceutical challenges. It belongs to the BCS Class II, exhibiting pH-dependent solubility and being practically insoluble at pH values above 6. This limits its solubilized presence in the small intestinal fluids with a pH of approximately 6.8 to 7.2, thus hindering absorption. Additionally, ACP is a substrate for the P-glycoprotein (P-gp) efflux transporter and is metabolized by the CYP3A enzyme system [16,17]. These factors contribute to its relatively low and highly variable oral bioavailability (25 ± 11%). There have been only a limited number of studies to enhance the solubility, dissolution rate, and oral bioavailability of ACP. Recent studies conducted by our team have demonstrated that reduction in the particle size of ACP (by formulating nanocrystals) significantly enhances its dissolution rate (>85% of the drug dissolving within 60 min), along with an increase in the absolute oral bioavailability (34.15 ± 9.02%), a notable improvement over the bulk drug [18]. In addition, Shettiwar and co-workers (2024) developed a lipid-based system using linalool that improved oral bioavailability (5.1-fold) in Balb/c mice [19].

In this study, we investigated the enhancement of the oral bioavailability of ACP by developing PLHNs loaded with ACP, utilizing various grades of mPEG-PCL in combination with suitable lipid(s). To gain a comprehensive understanding of the complex physical characteristics of PLHNs, we employed a Design of Experiments (DoE) approach rather than the traditional one-factor-at-a-time (OFAT) method. This robust approach allowed for the simultaneous evaluation of multiple factors and their interactions, as the physical properties of these complex carrier systems are interdependent and influenced by several material and manufacturing process parameters. The study also presents detailed in vitro dissolution, in vivo oral pharmacokinetics, and tissue distribution analysis, particularly towards the spleen. Our findings contribute to the expanding knowledge of NDDSs and provide valuable insights for the development of more effective treatments for conditions with limited therapeutic options.

## 2. Materials and Methods

### 2.1. Materials and Animal Models

ACP was requested as gift samples from MSN Laboratories Pvt. Ltd., Hyderabad, India, and prednisone (internal standard, IS, for the biomatrix-based HPLC analyses) was from Strides Pharma Pvt. Ltd., Hyderabad, India. For the conjugation reaction, ε-caprolactone (Monomer, Mw = 114.14 g/mol with 99.93% purity); mPEG (end functional group, M_w_ = 5000 g/mol); and stannous octoate (as the catalyst, Sn(Oct)_2_) were purchased from BLD Pharmatech India Pvt. Ltd., Hyderabad, India; Sigma Aldrich Pvt. Ltd., Mumbai, India; and Thermo Scientific Chemicals Pvt. Ltd., Hyderabad, India, respectively. For the preparation of nanoparticles, 1,2-dipalmitoylsn-glycero-3-phosphocholine (16:0 PC, LECIVA-DPPC) and lecithin USP NF (LECIVA-S90) were purchased from VAV life sciences Pvt. Ltd., Mumbai, India. Mannitol, Tween 80 (T80), and chloroform (CHCl_3_) were procured from Tokyo Chemical Industry Pvt. Ltd., Hyderabad, India. HPLC grade solvents, like toluene; dichloromethane (DCM); and diethyl ether, were purchased from SRL Pvt. Ltd., Mumbai, India. Isoflurane, an inhalational anesthetic, was sourced from Raman and Weil Pvt. Ltd., Mumbai, India. Milli-Q water, purified through an in-house Milli-Q water purification system (Merck Millipore, MA, USA), was used for all experimental procedures.

For the in vivo studies, male Wistar rats (weighing 220–250 g) were procured from Jeeva Life Sciences, Hyderabad, India (Registration number—1757/PO/ReBiBt/S/14/CPCSEA) following an assessment and approval of the in vivo experimental protocol (BITS-Hyd/IAEC/2022/15, Date of approval—23 April 2022) by the Institutional Animal Ethics Committee (IAEC).

### 2.2. Instruments and Software Systems

The conjugation reaction and preparation process of PLHNs involved a magnetic stirrer with temperature control (RCT-Basic, IKA India Pvt. Ltd., Bengaluru, India). PLHNs were prepared using a high shear homogenizer (Polytron PT3100D, Kinematica AG, Malters, Switzerland) fitted with a 12 mm standard dispersing aggregate (PT-DA 12/2EC-F154, Kinematica AG, Malters, Switzerland). The optimized product was freeze-dried using a freeze dryer (Coolsafe 110-4, LaboGene A/S, Allerød, Denmark). Chromatographic analysis of both in vitro and in vivo samples was conducted using a Shimadzu Prominence HPLC system (Shimadzu Corporation, Kyoto, Japan), which included a dual-pumping system (LC-20AD), a temperature-controlled autosampler (SIL-20ACHT), a column housing assembly (CTO-20AC), and a UV detector (SPD-M20A) with PDA amplification. The biomatrix-based samples were processed using a vacuum centrifugal concentrator (ScanVac) with an attached condenser (Coolsafe 110-4, LaboGene A/S, Allerød, Denmark).

Optimization of the critical factors influencing the critical response variables of PLHNs was conducted using a response surface methodology, designed with Design Expert software (version 13.0.5.0; Stat-Ease Inc., Minneapolis, MN, USA). In vivo pharmacokinetic data modeling was carried out using Phoenix WinNonlin software (version 8.4.0.6172; Pharsight Corporation, Mountain View, NC, USA).

### 2.3. Methods

#### 2.3.1. Preparation and Characterization of Conjugated Polymer(s)

##### Preparation of mPEG-Polycaprolactone (mPEG-PCL) Co-Polymer(s)

The mPEG-PCL co-polymer(s) was synthesized using the previously reported ring opening polymerization technique [10,20]. In a 25 mL borosilicate reaction tube (with a seal), mPEG (macroinitiator) and ε-caprolactone were taken in various molar ratios (1:9; 2:9; and 4:9), along with 10 mL of toluene (the reaction medium) and placed in an hot oil bath (previously maintained at 120 °C) under stirring at 100–110 rpm. Once the three components formed a clear solution, 0.01 mM Sn(Oct)_2_ (the catalyst) was gradually added to the reaction mixture to initiate polymerization, maintaining the aforementioned temperature and stirring speed. The reaction was carried out for 10–12 h at the said conditions until a viscous liquid was obtained. The obtained product was cooled down to room temperature (24 ± 1 °C) and dissolved in 20 mL of the solvent mixture (1:1 ratio) of dichloromethane and cold diethyl ether. The resultant homogenous mixture was subjected to concentration under vacuum using a rotary evaporator (Hei-VAP Core, Heidolph Instruments GmbH & Co., KG, Schwabach, Germany) to obtain a white precipitate. Once the precipitate was formed, the remaining supernatant solvent was discarded by decantation to remove the soluble impurities present in the supernatant. This process was repeated twice to obtain a conjugated polymer(s) (mPEG-PCL). The precipitate was then subjected to drying at 37 ± 2 °C under reduced pressure for 6–8 h to remove any traces of the residual solvents and obtain dry powder of the mPEG-PCL co-polymer(s).

##### Characterization of mPEG-PCL Co-Polymer(s)

To confirm the successful conjugation of the co-polymers, the chemical structure of the mPEG-PCL co-polymer(s) was determined by a proton nuclear magnetic resonance spectroscopy (^1^H NMR) (AV NEO, Bruker, Billerica, MA, USA with ASCEND™ 400 MHz/54 mm Long Hold Time Magnet operation field at 9.4 Tesla) in deuterated chloroform at 400 MHz and attenuated total reflectance Fourier transform infrared spectroscopy (ATR-FTIR) equipped with a diamond (Di) crystal material (Alpha II, Bruker, Billerica, MA, USA with Opus analysing software, Ver. 8.9). The thermal properties of the synthesized co-polymers were evaluated by differential scanning calorimetry (DSC) (TA-60 WS, Shimadzu, Kyoto, Japan) from 25 to 300 °C (at a rate of 10 °C/min).

The average Mw of the mPEG-PCL co-polymer(s) was determined by gel permeation chromatography (GPC) (Waters Corporation, Milford, MA, USA) equipped with a differential refractometric detector (Waters 2414 RI detector, Milford, MA, USA). A Styragel^®^ HR4 THF column (7.8 mm × 30 mm) (Waters, Milford, MA, USA) served as stationary phase, with tetrahydrofuran (THF) as the mobile phase (flow rate = 1 mL/min, injection volume = 20 µL, and sample concentration at 1 mg/mL across all the samples). The Mw of the synthesized co-polymers was analyzed using a standard regression equation obtained between the logarithm of the Mw of the polystyrene standards (5000; 9000; 17,500; 30,000; and 50,000 Da) (Sigma Aldrich Pvt. Ltd., Bengaluru, India) and their corresponding retention times.

#### 2.3.2. Preparation of Polymer Lipid Hybrid Nanoparticles

##### Preformulation Studies and Preliminary Trials

The ingredients used in the preparation of nanoparticles were selected based on their biocompatibility and ability to form nanoparticles with desired properties. The surfactants were chosen based on their ability to solubilize ACP and form stable nanoparticles with the desired particle size. The selection of lipids (lecithin S90, di-stearoyl phosphatidylcholine (DSPC), di-stearoyl phosphatidylglycerol sodium salt (DSPG), and DPPC) was driven by their functional properties and their capacity to form PLHNs with optimal characteristics, including particle size (PS), the polydispersity index (PDI), loading efficiency (LE) or entrapment efficiency (EE), and stability.

To identify the critical material attributes (CMAs) and critical process parameters (CPPs) affecting the PS, PDI, LE, and EE of the nanoparticles, a series of preliminary formulation trials were conducted. Based on the number of CMAs and CPPs, an appropriate experimental design was selected, either a hybrid design (screening followed by optimization) or an optimization design using response surface methodology (RSM), using Design Expert software.

##### Implementation of DoE

For both formulations, acalabrutinib-loaded lipophilic and hydrophilic PLHNs (*l*ACP-PLHNs and *h*ACP-PLHNs), involving two different grades of mPEG-PCL, the critical factors influencing the PS, PDI, LE, and EE were kept same - the ratio of mPEG-PCL to lipid, the concentration of T80, the homogenization speed, and duration. Further, for a comparative evaluation, the factorial levels were also set at the same values for both formulations. An RSM-based design was used to optimize the preparation of both the formulations, as there were only 4 critical factors affecting the responses. A spherical and rotatable circumscribed central composite design (cCCD) with star/axial points, with the ‘α’ value set at 2, was selected as the RSM to optimize the factors. As shown in Table 1, the factors were evaluated at five levels (−α, −1, 0, +1, and +α). The software recommended a total of 28 experimental runs, comprising 16 factorial runs (no replications), 8 α-point runs (no replications), and 4 replications of the center point runs.

The best-fit regression model for each response was determined using ANOVA at a significance level of 0.05 to assess model significance and potential lack of fit. Additionally, diagnostic plots, such as Box–Cox power transformation plots, residual plots, and predicted versus actual plots, were used to assess the goodness-of-fit and the impact of the transformations. To further understand the influence of the most significant factors on PS, PDI, LE, and EE, 3D response surface plots were generated.

##### Method of Preparation

**Figure 1:**
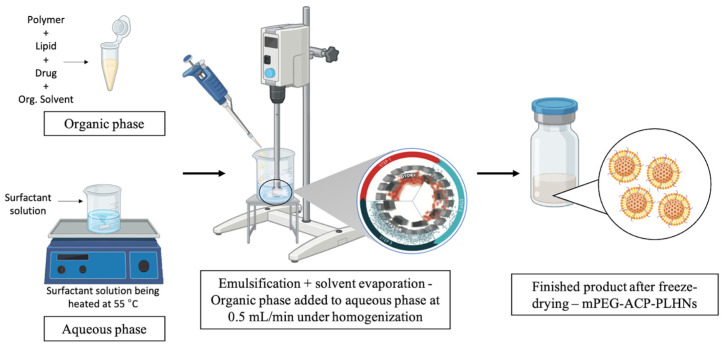
A schematic diagram illustrating the manufacturing process of both types of ACP-PLHNs along with the anticipated structure of the ACP-PLHNs.

Both types of ACP-PLHNs were prepared using the emulsification solvent–evaporation method with a high-shear homogenizer [21,22] (Figure 1). For the preparation of *l*ACP-PLHNs, the organic phase (OP) contained 20 mg of ACP, with the conjugated polymer and lipids in a 1.711:1 ratio. This mixture included 17.11 mg of mPEG-PCL (lipophilic grade) and 5 mg each of DPPC and lecithin dissolved in 1000 μL of CHCl_3_. The aqueous phase (AP) was prepared by dissolving 0.806% *w*/*v* T80 in Milli-Q water and stirring at 250 rpm while maintaining a temperature of 55 °C. The AP was then homogenized at 16,600 rpm, and the OP (containing ACP, the conjugated polymer, and lipids in a solubilized state) was added to the AP in a controlled fashion (0.5 mL/min) under ambient conditions (23–25 °C). The total processing time was 15 min. In contrast, for the preparation of *h*ACP-PLHNs, the OP was composed of 20 mg of ACP, along with the polymer and lipids in a 2.487:1 ratio. Specifically, 24.87 mg of mPEG-PCL (hydrophilic grade) and 5 mg each of DPPC and lecithin were dissolved in 1000 μL of CHCl_3_. The AP consisted of 0.876% *w*/*v* of T80 in Milli-Q water and was prepared under the same conditions as the *l*ACP-PLHNs. The AP was homogenized at 17,000 rpm, into which the OP was added. Homogenization was continued for 19 min at 17,000 rpm to break down the globules, evaporate the CHCl_3_, and form PLHNs with the desired PS. Both formulations were left to rest for 60 min at room temperature (24 ± 1 °C) to ensure maximum removal of CHCl_3_. For freeze-drying, the formulations were individually centrifuged at 16,627× *g* at 10 °C for 30 min. The resulting pellet was washed twice with 10 mL of 0.25% *w*/*v* T80 solution and centrifuged under the same conditions. After the final wash, the pellet was re-dispersed in a 0.25% *w*/*v* T80 solution containing 2% *w*/*v* mannitol, a cryoprotectant, and then freeze-dried using a freeze dryer.

#### 2.3.3. Method of Analysis Using HPLC-UV

In vitro and in vivo samples were analyzed using a previously optimized and validated HPLC-UV method by our research group [18]. ACP was quantified using a Phenomenex Luna^®^ Omega polar C18 column (150 mm × 4.6 mm, 5 µm, Phenomenex Inc., Torrance, CA, USA) as the stationary phase and 10 mM ammonium acetate buffer (pH 3.5) and methanol as the mobile phase. For in vitro analysis, the column was maintained at 55 °C, the mobile phase ratio was 48:52 (aqueous/organic), the flow rate was 1.05 mL/min, and the detection wavelength was maintained at 250 nm. The retention time of ACP was 4.24 ± 0.018 min, with linearity from 0.05 to 5 µg/mL, an LOD of 1.46 ng/mL, and an LOQ of 4.32 ng/mL.

To quantify the drug in the plasma or spleen samples obtained from the in vivo studies, ACP was extracted using acidified methanol (1% formic acid), evaporated, and reconstituted in the mobile phase (60:40 of aqueous/organic). The column temperature was 53 °C, with a flow rate of 1.1 mL/min. ACP and IS had retention times of 5.96 ± 0.03 and 8.29 ± 0.02 min, respectively. The method showed linearity from 0.08 to 5 µg/mL, with an LOD and an LOQ of 74.53 ng/mL and 24.84 ng/mL, respectively.

#### 2.3.4. Characterization of Optimized ACP-PLHNs

##### Determination of PS, PDI, and Surface Charge

Optimized formulations of both *l*ACP-PLHNs and *h*ACP-PLHNs were subjected to evaluation of the PS, PDI, and zeta potential using a zeta sizer (Zeta sizer NANO ZS, Malvern Pananalytical Ltd., Worcestershire, UK). PS and PDI were measured based on the principle of dynamic light scattering, while zeta potential was measured based on electrophoretic light scattering. Both freshly prepared and reconstituted freeze-dried ACP-PLHNs (1 mg powder/mL of filtered Milli-Q water dispersed by vortex mixing for 5 min) were diluted 150× with Milli-Q water and placed in a disposable polystyrene cuvette for analysis. Each sample was measured in triplicate, with 15 iterations per measurement, under conditions including a 120 s equilibration time and a temperature of 25 °C. The samples were exposed to a He-Ne laser (4.0 mW, 633 nm) with detection at a backscatter angle of 173°. Zeta potential was determined using a folded capillary zeta cell, employing the Smoluchowski model and a Debye function (F(κa)) value of 1.50.

##### Morphological Evaluation

The shape of both types of optimized ACP-PLHNs was evaluated using an atomic force microscope (AFM, CoreAFM, Nanosurf AG, Liestal, Switzerland) equipped with a CoreAFM control software ver. 3.10. Reconstitution of the freeze-dried ACP-PLHNs was achieved using filtered Milli-Q water (1 mg powder/mL) spread uniformly on a glass slide. The layer was gradually dried at room temperature for 10–12 h, followed by terminal drying at a reduced pressure (temperature 30 °C) for 60 min. The resultant dried layer was evaluated by a tapping mode with the scan parameters set at 1 s/line; 256 points/line; and a rotation of 0°.

##### Loading Efficiency (LE) and Entrapment Efficiency (EE)

The freshly prepared nanosuspensions were subjected to both indirect and direct methods for the estimation of LE and EE. The nanosuspensions were centrifuged at 16,627× *g* at 10 °C for 30 min. The supernatant, expected to contain free ACP, was collected for indirect measurement. The pellet was washed twice with 5 mL of Milli-Q water and then dissolved in 2 mL of methanol for direct measurement. Both the supernatant (for indirect measurement) and dissolved pellets (for direct measurement) were diluted with the mobile phase (48 parts of ammonium acetate buffer solution and 52 parts of methanol) and analyzed via HPLC-UV, following the method described in Section 2.3.3. The LE and EE were calculated using Equations (1) and (2):

(1) $$\mathrm{LE}\;\left(\%\right)=\left(\frac{{\mathrm{Total}\;\mathrm{amt}}_{\mathrm{ACP}\;}-{\;\mathrm{Amt}}_{\mathrm{free}\;\mathrm{ACP}}}{{\mathrm{Total}\;\mathrm{amt}}_{\mathrm{ACP}}+{\;\mathrm{Total}\;\mathrm{amt}}_{\mathrm{lipid}+\mathrm{polymer}}}\right)\times 100$$

(2) $$\mathrm{EE}\;\left(\%\right)=\left(\frac{{\mathrm{Total}\;\mathrm{amt}}_{\mathrm{ACP}}-{\;\mathrm{Amt}}_{\mathrm{free}\;\mathrm{ACP}}}{{\mathrm{Total}\;\mathrm{amt}}_{\mathrm{ACP}}}\right)\times 100$$

where ${\mathrm{Total}\;\mathrm{amt}}_{\mathrm{ACP}}$ = ACP added per batch of the formulation; ${\mathrm{Amt}}_{\mathrm{free}\;\mathrm{ACP}}$ = the amount of ACP, in free form, present in the supernatant; and ${\mathrm{Total}\;\mathrm{amt}}_{\mathrm{lipid}+\mathrm{polymer}}$ = the amount of DPPC, lecithin, and mPEG-PCL added per batch of the formulation. All measurements were performed and reported for three independent batches of the optimized formulations. As there was no significant difference between the values obtained from the direct and indirect methods of analysis, the indirect method was selected for further calculations of LE and EE.

##### Thermal Analysis

Thermal analysis was performed using a DSC for pure ACP, a physical mixture of the two grades of mPEG-PCL individually with lipids (DPPC + lecithin) (1:1 ratio), pure mannitol, and freeze-dried placebo and ACP-PLHNs. Samples weighing approximately 3–4 mg were placed in aluminum pans and crimped using a hand press. For pure ACP, an amount corresponding to the drug content in the freeze-dried formulation(s) was used. The reference (blank crimped aluminum pan) and sample pans were placed in the DSC chamber, acclimated to 30 °C, and purged with nitrogen at 50 mL/min. After equilibration, the samples were analyzed from 30 °C to 300 °C (at 10 °C/min) under nitrogen purging. The thermograms were analyzed using TA-60 software (Ver. 1.5).

##### Powder X-Ray Diffraction (P-XRD)

To assess the physical nature of the entrapped ACP, pure ACP, a physical mixture of two grades of mPEG-PCL, individually mixed with the lipids (DPPC + lecithin) (1:1 ratio), and freeze-dried ACP-PLHNs were analyzed using p-XRD (ULTIMA IV Rigaku, The Woodlands, TX, USA) equipped with a scintillation counter detector. The measurements were performed in 2θ mode over a range of 10° to 80°, using a copper X-ray source, a scanning speed of 3°/min, and a temperature of 25 °C. Data analysis was conducted using PDXL2 software (Ver. 2.9).

##### Estimation of Residual Solvents

The preparation of ACP-PLHNs involved the use of CHCl_3_ as the solvent, and the presence of residual solvent was assessed using a capillary gas chromatograph (GC-2010 plus, Shimadzu Corporation, Kyoto, Japan) with a flame ionization detector. A Spinco Tech Enable EB-1 column (Spinco Tech Pvt. Ltd., Chennai, India) with dimensions 30 m × 0.25 mm and a film thickness of 0.25 µm was used as the stationary phase. The temperature program started at 40 °C, was held for 1 min, followed by a 30 °C/min ramp to 120 °C, was held for 5 min, followed by a 30 °C/min ramp to 210 °C, and was held for 1 min. The mobile phase consisted of a nitrogen–air mixture, with 54.6 mL/min as the total flow rate and 1.01 mL/min as the column flow rate in a split mode (1:50 ratio). The injection port and detector were maintained at 220 °C and 250 °C, respectively. Dimethyl sulfoxide (DMSO) was used as the solvent, in which both pure CHCl_3_ and both types of freeze-dried ACP-PLHNs (3 mg each in 1000 µL) were solubilized. A calibration curve for CHCl_3_ was created in the range of 1.5 to 120 ppm (10 to 800 µL/mL). The retention time of CHCl_3_ was around 2.7 min, with a total run time of 13.67 min.

##### In Vitro Drug Release Studies

A membrane-less method using a USP Type II dissolution apparatus (Manual operating model with 8 stations, Electrolab (India) Pvt. Ltd., Mumbai, India) was employed to evaluate the dissolution and drug release behavior of both bulk ACP and freeze-dried ACP-PLHNs (both types, equivalent to 100 mg free base) under physiological pH conditions. The gastrointestinal pH was simulated with three different buffer solutions: a 0.1 N HCl buffer (pH 1.2 ± 0.02, volume = 250 mL) representing the fasted stomach; an acetate buffer (pH 4.5 ± 0.02, volume = 250 mL) representing the fed stomach; and a phosphate buffer solution (PBS, pH 6.8 ± 0.02, volume = 900 mL) representing the intestinal environment. To simulate blood/plasma conditions, 500 mL of PBS (pH 7.2 ± 0.02) was used. To maintain sink conditions, 0.5% *w*/*v* sodium dodecyl sulfate (SDS) was added to media with pH ≥ 4.5, and the volume of medium was kept at 3× the volume required to maintain the saturation solubility of ACP. The formulations were dispersed in the dissolution media, which was continuously stirred at 75 rpm and maintained at 37 ± 2 °C. Samples (1000 μL) were withdrawn at specific time intervals and replaced with an equal volume of fresh media (pre-heated to 37 ± 2 °C). The samples were then centrifuged at 15,373× *g* for 5 min, and the supernatant was collected, diluted with the mobile phase (methanol and a 10 mM ammonium acetate buffer, pH 3.5, in a 52:48 *v*/*v* ratio), and analyzed using the HPLC-UV method outlined in Section 2.3.3. The dissolution data were fitted to different mathematical models to determine the drug release kinetics. The best-fitting model was identified based on the regression coefficient.

##### Stability Studies

The impact of storage conditions on freeze-dried ACP-PLHNs was assessed following ICH Q1A(R2) guidelines. The product was stored in sealed glass vials (n = 3) under three conditions: 45 ± 2 °C/75 ± 5% relative humidity (RH); 25 ± 2 °C/60 ± 5% RH; and 5 ± 2 °C for 6 months. Critical attributes (PS, PDI, and EE) were monitored at regular intervals. The results are presented as mean ± SD for PS and PDI, and percentage deviation for EE relative to the fresh formulations.

##### Preventive Evaluations

Prior to in vivo evaluation, the safety of the optimized formulations in terms of colloidal stability, hemolytic potential, and their effect on the morphology of red blood cells (RBCs) was assessed.

##### Colloidal Stability

To assess colloidal stability, freeze-dried ACP-PLHNs, both formulations (~10 mg ACP), were dispersed in 80 mL of pH 7.2 ± 0.02 buffer and maintained at 37 ± 2 °C with continuous stirring at 75 rpm. Samples were withdrawn at various time points over a 24 h period, and aggregation was evaluated by measuring the PS. The measurement procedure followed the method described in Section 2.3.4 (Determination of PS, PDI, and surface charge).

##### Hemolytic Potential

RBCs were collected from healthy male Wistar rats and dispersed as a 2% *v*/*v* suspension using a 0.9% sodium chloride solution. Both freeze-dried *l*ACP-PLHNs and *h*ACP-PLHNs were reconstituted with Milli-Q water to concentrations of 1, 2.5, 5, 7.5, and 10 mg/mL ACP. Aliquots (100 μL) from each suspension were added to 900 μL of RBC–saline suspension, resulting in final ACP concentrations of 0.1, 0.25, 0.5, 0.75, and 1.0 mg/mL. Saline and 1% Triton X were used as negative and positive controls, respectively. Samples were incubated at 37 ± 2 °C for 1 h and then centrifuged at 3500 rpm for 10 min. Hemolysis was determined by measuring the absorbance of the supernatant at 545 nm, as per Equation (4):

(3) $$\%\mathrm{Hemolysis}=\frac{{\mathrm{Abs}}_{\mathrm{sample}}-{\;\mathrm{Abs}}_{-\mathrm{ve}\;\mathrm{control}}}{{\mathrm{Abs}}_{+\mathrm{ve}\;\mathrm{control}}-{\;\mathrm{Abs}}_{-\mathrm{ve}\;\mathrm{control}}}\times 100$$

where ${\mathrm{Abs}}_{\mathrm{sample}}$ = the absorbance of the samples incubated with different concentration of ACP loaded in either *l*ACP-PLHN and *h*ACP-PLHN carrier systems; ${\mathrm{Abs}}_{-\mathrm{ve}\;\mathrm{control}}$ = the absorbance of the samples incubated with a negative control; and ${\mathrm{Abs}}_{-\mathrm{ve}\;\mathrm{control}}$ = the absorbance of the samples incubated with a positive control. In each condition and for each treatment/formulation, the measurements were recorded in triplicate.

##### Effect on RBC Morphology

The reconstituted freeze-dried ACP-PLHNs (both optimized formulations) were orally administered (dose = 30 mg/kg, dosing volume = 4 mL/kg) to healthy male Wistar rats (n = 3, ~220 g), and the blood samples were collected at 0.5, 0.75, and 1 h post-dosing to evaluate the effect of the nanoparticle formulations on the morphology of RBCs. From each of the blood samples collected, the RBCs were separated by centrifugation (3500 rpm, 10 min, 4 °C) and resuspended in cold PBS (pH 7.2) to form a 2% *v*/*v* suspension. The suspension containing RBCs was treated with 1% *v*/*v* Triton X (the positive control for lysis) or 0.9% *w*/*v* saline (the negative control). The RBCs were fixed with 2% *v*/*v* glutaraldehyde solution, incubated at 4 °C in the dark for an hour, and washed with cold PBS. The cells were dehydrated through increasing ethanol concentrations (30%, 50%, 80%, 90%, and 100% *v*/*v*) for 15 min each and then rehydrated in cold Milli-Q water. A small amount of the final suspension was drop-cast onto a silicon wafer and allowed to dry at 20 ± 2 °C for 10–12 h. The dried sample was sputter-coated (with a gold–aluminum mixture) and analyzed using FE-SEM at various magnifications.

##### In Vivo Oral Pharmacokinetics (PK) and Tissue Distribution Study

After procurement, the animals were quarantined for 15 days under ambient conditions (12 h light/dark cycle, 22 ± 1 °C, 50 ± 10% RH) with unrestricted access to rat chow and water. Prior to treatment, the rats were weighed, randomly assigned to treatment groups, and transferred to clean cages for a 10–12 h fasting period, during which they had free access to water only, to prevent drug–food interactions and coprophagia.

##### Single Oral PK Studies

A total of 6 animals were randomly assigned to 2 treatment groups—*l*ACP-PLHNs and *h*ACP-PLHNs—with n = 3 animals per treatment group. Both types of ACP-PLHN nanosuspension were reconstituted from freeze-dried powder with Milli-Q water and uniformly dispersed by vortex mixing, followed by sonication. Both formulations were administered at a dose of 30 mg/kg (4 mL/kg volume) via an oral feeding needle (16G × 50 mm). Blood (300 µL) was collected from the retro-orbital plexus at pre-fixed time points (pre-dose, 0.88, 1.67, 0.42, 0.75, 1, 2, 4, 6, 8, 10, 12, and 24 h) into tubes containing 10% *v*/*v* anticoagulant (40 mg/mL disodium EDTA). Plasma samples were processed and analyzed, as described in Section 2.3.3. To assess the absolute and relative bioavailability of both types of ACP-PLHN nanosuspension, the PK data of the intravenous bolus administration of the simple ACP solution (12 mg/kg) and oral administration of the conventional ACP suspension (30 mg/kg) from previously reported data by our research group was used in the analysis [18]. The PK parameters were determined by analyzing plasma concentration–time data using regression analysis based on the linearity range with Phoenix WinNonlin software.

##### Drug Distribution Studies on Spleen

To quantify ACP levels in the spleen, n = 4 healthy male Wistar rats were used for both optimized formulations of ACP-PLHNs. The freeze-dried ACP-PLHN nanosuspension was administered orally at the same dose used in the pharmacokinetic (PK) studies. Spleen samples were collected at two specific time points: T_max_ of the conventional ACP suspension (0.75 h) and T_max_ plus twice the half-life of the drug in rats (3.75 h). At each time point, n = 2 rats were euthanized for spleen collection. Adipose tissue was carefully removed using forceps, and the spleens were cleaned in cold PBS and dried with tissue paper. Each spleen was weighed and minced separately, followed by suspension in cold, filtered PBS (3 mL per g of tissue). Further, they were homogenized at 7300–8000 rpm using a tissue homogenizer (T10 basic ULTRA-TURRAX^®^, IKA India Pvt. Ltd.). To extract the ACP and IS, a protein precipitation method was adopted using 800 μL of acidified methanol. The supernatant was collected after centrifugation (14,167× *g*, 8 °C for 12 min) and concentrated using a vacuum concentrator (1200 rpm, 10 °C for 4 h). The dried mass was reconstituted in 100 μL of a mobile phase (a 40:60 *v*/*v* methanol/ammonium acetate buffer, pH 3.5), mixed thoroughly, and centrifuged (14,167× *g*, 8 °C for 10 min). The clear supernatant was then analyzed by HPLC-UV using the bioanalytical method described in Section 2.3.3.

## 3. Results

### 3.1. Preparation and Characterization of Different Grades of mPEG-PCL Co-Polymer(s)

Two different grades of mPEG-PCL, using different molar ratios of mPEG and ε-caprolactone, were prepared using a well-established method of ring-opening polymerization reaction, as detailed in Section 2.3.1. In the reaction, mPEG and ε-caprolactone were used in the ratio of 1:9 and 4:9 to synthesize the lipophilic and hydrophilic grades of the co-polymers, respectively. The terms “lipophilic” and “hydrophilic” are used depending on the extent of the solubility of the respective co-polymers in Milli-Q water (pH = 7 ± 2) in benchtop conditions (25 ± 2 °C). Two different grades of the co-polymer were synthesized to evaluate their effect on the formulation of PLHNs loaded with the hydrophobic drug, ACP. This ideation was supported by a study performed by Mohanty A et al., 2014, which reported that a variation in the molar ratio between mPEG and ε-caprolactone affects the ability of the resulting polymer to encapsulate a hydrophobic moiety and the kinetics of its release [23]. Both co-polymers were white to off-white in color and odorless. They each had a waxy finish, with the lipophilic grade appearing more granular compared to the hydrophilic grade.

**Figure 2:**
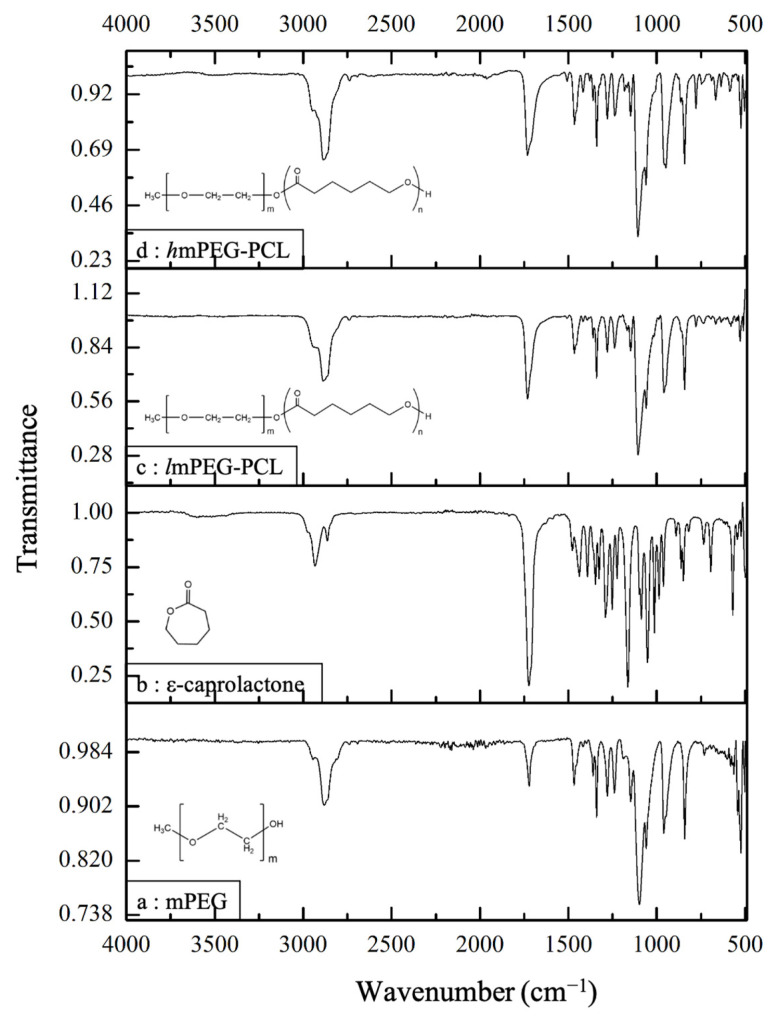
The FTIR spectrum of the reaction components and co-polymers: mPEG (**a**) and ε-caprolactone (**b**); and conjugated lipophilic (**c**) and hydrophilic (**d**) mPEG-PCL co-polymers.

To ascertain a successful conjugation between mPEG and ε-caprolactone, an ATR-FTIR was performed. The bands with varying intensities around 3000–2840 cm^−1^ in Figure 2a,b correspond to the “C–H” stretching vibrations of the aliphatic “–CH_2_–“ and “–CH_3_” groups present in both mPEG and ε-caprolactone. A very strong band near 1100–1150 cm^−1^ (Figure 2a) is characteristic of the ether linkages (–OCH_2_–) in the mPEG chain, while a strong band near 1735–1750 cm^−1^ (Figure 2b) corresponds to the lactone carbonyl group (C=O) of ε-caprolactone. The “C–O” stretch in the methoxy group of mPEG, as well as the “C–O–C” stretch from the ether linkages in the PEG backbone, are represented by bands around 1000–1050 cm^−1^ (Figure 2a). For ε-caprolactone, the bands near 1050–1150 cm^−1^ and 1300–1450 cm^−1^ correspond to the “C–O” stretch from the ester linkage and “C–H” bending (scissoring) vibrations of the alkane chain, respectively. In Figure 2c,d, the intense bands around 1100–1200 cm^−1^ form an overlapping region for both the ester bond in PCL and the ether linkage (–OCH_2_–) in mPEG. The band around 1725–1750 cm^−1^ represents the characteristic ester carbonyl (–COO–) functional group in the PCL repeating units of the co-polymer. The presence of aliphatic “–CH_2_–” and “–CH_3_” groups in both mPEG and PCL is indicated by the bands around 2800–2950 cm^−1^. Notably, the peak intensity in this region is comparatively stronger for the mPEG-PCL co-polymer, suggesting a higher number of alkane groups corresponding to the greater number of mPEG units in the co-polymer. Similar spectra for the mPEG-PCL co-polymers have also been reported by Danafar H et al., 2014 [20] and Hemmati K et al., 2016 [24].

**Figure 3:**
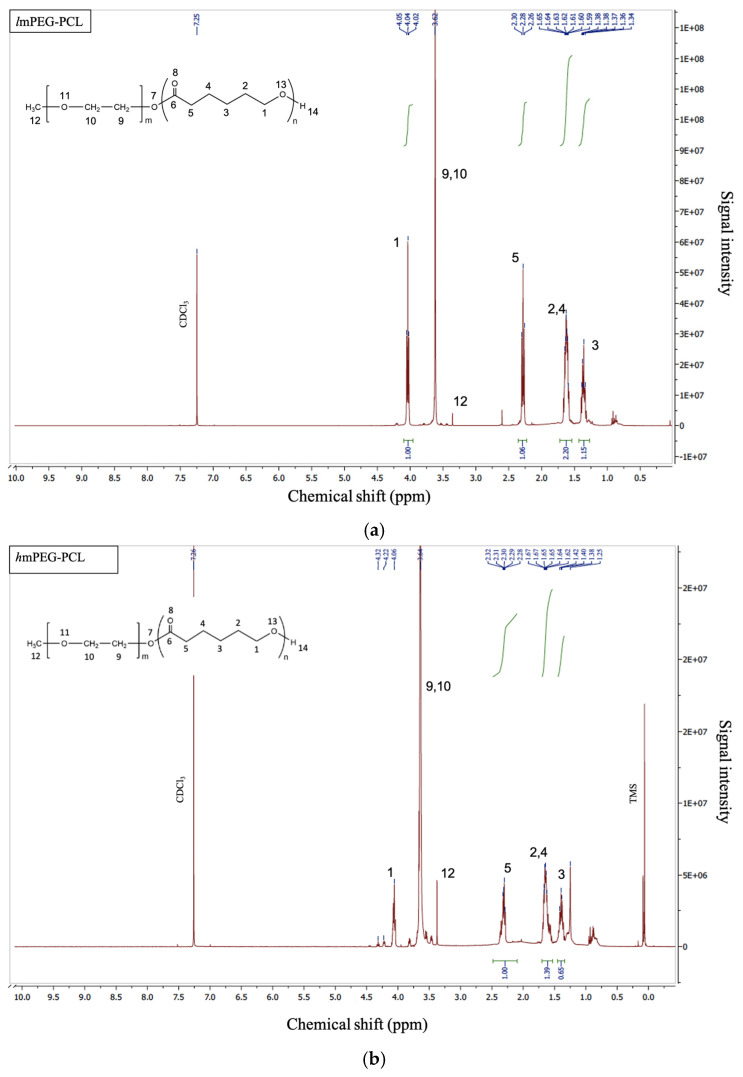
NMR spectrum of one block co-polymer of lipophilic mPEG-PCL (**a**) and hydrophilic mPEG-PCL (**b**) dissolved in CDCl_3_, where m = 1 and n = 1.

Further, the ^1^H NMR spectrum of both grades (dissolved in CDCl_3_) (Figure 3) shows that peaks at 2.0–2.5 ppm representing the multiplet are due to the methylene protons adjacent to lactone carbonyl in PCL, while the singlet at around 3.5 is due to the protons of the two -CH_2_ groups adjacent to the methoxy group of m-PEG, thereby confirming the structure of the obtained conjugated polymers. In the case of *h*mPEG-PCL (Figure 3b), the intensity of the singlet peak around 3.5 is higher due to the presence of a higher molar ratio. The small peak around 3.3 represents the terminal protons of the mPEG’s methoxy (-OCH_3_) group. The peaks (multiplets) around 4.0–4.5 ppm are due to the methylene protons adjacent to the terminal -OH group in PCL. Earlier research works involving the synthesis of mPEG-PCL block co-polymers have reported similar peaks representing successful conjugation [10,20,25,26].

Finally, based on the GPC analysis using the polystyrene standards (and the regression equation, $y=0.0182\;x^{3}-0.4805\;x^{2}+3.0115\;x+10.763$ with $R^{2}=0.994$), the molecular weights of the lipophilic and hydrophilic mPEG-PCL were found to be 9817.67 and 23,615.84 Da, respectively, with a PDI of 1.22 and 1.18, respectively. In polymerization reactions, as per the literature reports, PDI values in the range of 1.0 to 1.2 indicate a well-controlled catalysis, resulting in a narrow molecular weight distribution of the polymer [23,27]. Furthermore, the PDI of 1.22 for lipophilic mPEG-PCL is considered realistic and indicates controlled polymerization [28].

### 3.2. Preformulation Studies

#### 3.2.1. Selection of Formulation Components

The surfactant that exhibited the lowest solubility for ACP (even at higher surfactant concentrations) was selected as the most suitable stabilizer for the preparation of ACP-PHLNs. This choice was based on the principle that a surfactant in which the drug has low solubility will minimize the amount of drug partitioning from the organic phase into the aqueous phase during the preparation of the nanoparticles by the emulsification–solvent evaporation technique. Among the surfactants tested (referred from the previously reported solubility trials of ACP in different concentrations of surfactants by the same research group), poloxamer 188 (P188), T80, and polyvinyl alcohol (PVA) met this criterion [29]. Further, the most suitable surfactant was chosen based on the physical characterization (PS, PDI, and stability) of the ACP-PLHNs. In the current research, when using P188, both types of formulations exhibited an average PS of 215.28 ± 28.37 nm, with a PDI of 0.236 ± 0.15. In contrast, using T80 and PVA, the average PS was 168.21 ± 10.9 nm and 383 ± 6.21 nm, with a PDI of 0.210 ± 0.03 and 0.382 ± 0.40, respectively. Further, T80 offered a stability of 3 days at 24 ± 1 °C compared to P188 and PVA. Amongst the three lipids, DPPC was chosen because of the physical characters (PS, PDI, EE, and stability) offered to the formulation (PS = 174.93 ± 7.12 nm, PDI = 0.225, and EE = 73.82% stable for 3 days at 24 ± 1 °C) compared to the other lipids. In order to enhance the storage stability of the freshly prepared formulation in benchtop conditions (24 ± 1 °C), lecithin and cholesterol were incorporated, along with DPPC. Among these two, lecithin enhanced the stability of the freshly prepared PLHNs from 3 days to 6 days (with no apparent change in PS over a period of 6 days).

Thus, lipophilic and hydrophilic grades of in-house conjugated mPEG-PCL were selected as the polymers, which were individually combined with DPPC and lecithin to prepare *l*ACP-PLHNs and *h*ACP-PLHNs, respectively. In the preparation of the nanoparticles, CHCl_3_ (organic phase) was used to dissolve the polymer, lipid, and drug. Meanwhile, an aqueous solution of T80 (prepared using filtered Milli-Q water) was used as the aqueous phase.

#### 3.2.2. Selection of Critical Response Variables, Critical Factors, and Factorial Levels

An ideally designed nanoparticle should possess optimized physical properties such as PS, zeta potential, and shape to evade clearance by the mononuclear phagocytic system, remain in circulation long enough for targeted tissue accumulation, and be internalized by the target tissue in order to enhance therapeutic efficacy [30,31]. In the current nanoparticle formulation, the stabilizer (T80) used was a steric stabilizer rather than an electrostatic stabilizer. Further, the remaining ingredients were also non-ionic in nature and did not contribute to the zeta potential of the nanoparticles. Therefore, the resulting PLHNs were expected to exhibit a near-neutral zeta potential. The morphological analysis of a few preliminary batches (prepared with different sets of excipients) revealed that the nanoparticles were only of spherical shape. Hence, the zeta potential and shape of the nanoparticles were not affected by any excipients or processing parameters, and thereby, these were not considered the critical response variables (CRVs) for the current formulations. Additionally, LE and EE are key factors that indicate the drug amount per unit weight of the formulation and the proportion of drug successfully encapsulated within the nanoparticles, respectively. Both parameters can be determined using similar mathematical equations and are influenced by the type and amount of excipients, along with the manufacturing conditions. EE is further influenced by the mode of separation of the free drug from the nanoparticles. Hence, for the formulation of both *l*ACP-PLHNs and *h*ACP-PLHNs, PS (Y_1_) and EE (Y_2_) were considered to be CRVs.

Two energy-dependent preparative methods were employed for the preparation of PLHNs: a “two-step process” where the primary O/W emulsion was first prepared through temperature-dependent high-speed stirring, followed by globule/particle breakdown and CHCl_3_ evaporation using probe sonication (ultrasonication); and a “one-step process”, where emulsification, size reduction, and CHCl_3_ evaporation occurred simultaneously through high shear homogenization (HSH). The PS for both *l*ACP-PLHNs and *h*ACP-PLHNs prepared using ultrasonication was >300 nm, with a PDI of >0.43. In contrast, the PS of both formulations, when prepared using high shear homogenization, was <250 nm, with a PDI of <0.3. Hence, the “one-step process” using high shear homogenization was adopted as the method of preparation for both types of formulation. Table 2 enlists the various material- and process-related factors affecting the CRVs of both types of PLHNs.

Apart from the listed parameters in Table 2, the temperature of the AP, when subjected to homogenization, was crucial and impacted the PS. When the AP was maintained at 24 ± 1 °C, the PS was within 180 to 200 nm, while at 54 ± 1 °C, the PS was within 150 to 170 nm. Neither the rate of addition of OP to AP nor the ratio of the volume of OP to AP affected the CRVs of the formulations. Hence, for both formulations, the temperature of AP; the rate of addition of OP to AP; and the ratio of the volume of OP to AP were fixed at 55 °C; 0.5 mL/min; and 1:25, respectively. Finally, critical factors, including the ratio of mPEG-PCL to lipid (A), concentration of T80 (B), homogenization speed (C), and homogenization duration (D), were taken for optimization using DoE.

### 3.3. Implementation of DoE

Given the fact that there were only four factors affecting the CRVs and the complexity and cost involved, conducting separate screening experiments was deemed unnecessary. As a result, the four factors were directly incorporated into the RSM optimization design. A cCCD was selected, with an axial distance (α) of 2, ensuring both sphericity and rotatability for the design. A total of 28 experimental runs were carried out in a single block, as detailed in Table 3 and Table 4. This approach was feasible because the raw materials (including their grade and source), environmental conditions, and laboratory equipment were consistent throughout all the experiments for both formulations.

#### 3.3.1. Effect on PS (Y_1_)

**Figure 4:**
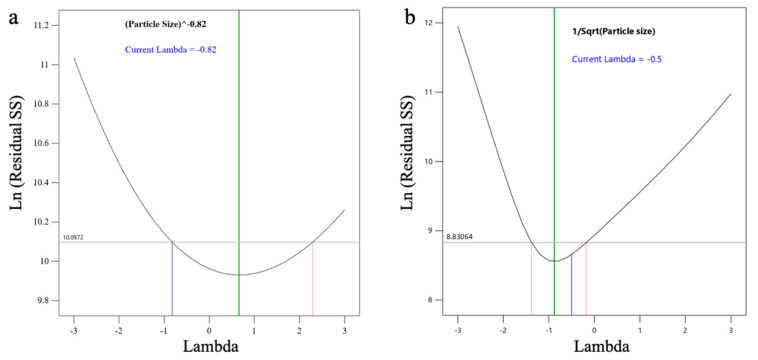
Box–Cox power plots showing the best transformation function for PS (Y_1_) of *l*ACP-PLHNs (**a**) and *h*ACP-PLHNs (**b**).

For *l*ACP-PLHNs, a reduced two-factor interaction model (with appropriate model significance) was chosen based on the removal of the model terms with *p* > 0.1, along with maintaining an intact model hierarchy. The PS was most affected by the interaction between the ratio of mPEG-PCL to lipid (A) and the concentration of T80 (B). In contrast, the PS of the *h*ACP-PLHNs was significantly influenced by both T80 concentration (Factor B) and homogenization speed (Factor C). The goodness-of-fit for both ACP-PLHN models can be assessed from the corresponding Box–Cox power transformation plots, which illustrate how well the data conforms to the assumed transformation function for PS (Figure 4). The regression equation for PS, in a coded form, for *l*ACP-PLHNs and *h*ACP-PLHNs are stated as Equations (5) and (6), respectively.

(4) $${\mathrm{PS}}^{\left(-0.82\right)}=0.0178-0.0012\;A\;+0.0002\;B\;+0.0014\;C\;-0.0017\;\mathrm{AB}\;$$

(5) 1PS=0.0696−0.0009 A+0.0047 B+0.0176 C−0.0035 D+0.0015 AB+0.0043 AC+0.0042 BD+0.0016 A2+0.0037 B2+0.0095 C2−0.0022 D2−0.0167 A2C+0.0054 AB2

**Figure 5:**
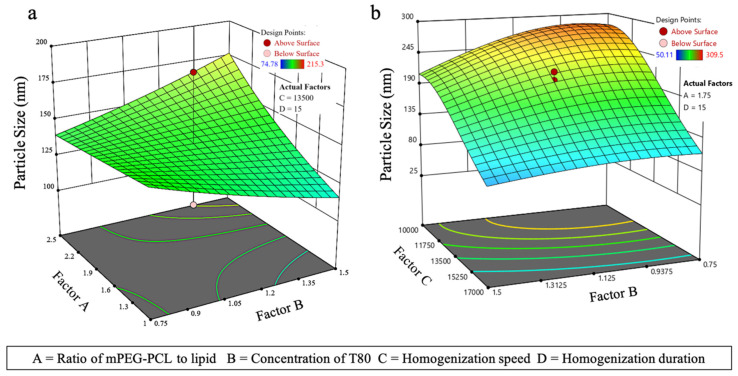
Three-dimensional response surface plots for PS (Y_1_) of *l*ACP-PLHNs (**a**) and *h*ACP-PLHNs (**b**).

The 3D plots for both formulations, showing the relationship between PS and the influencing factors, display a complex topography. To better understand the underlying effects, these plots can be divided into distinct segments for clearer analysis. Figure 5a displays a twisted surface, which is attributed to the interaction between the ratio of mPEG-PCL to lipid and the concentrations of T80. The PS increases when both the mPEG-PCL and T80 concentrations are higher. This can be explained by two factors: First, the increased concentration of mPEG-PCL and T80 raises the overall viscosity of the formulation, resulting in slower homogenization or insufficient shear force from the homogenizer. This promotes the formation of larger aggregates rather than smaller nanoparticles. Second, when the mPEG-PCL concentration is higher, the amount of T80 may be insufficient to adequately reduce the interfacial tension, leading to a higher likelihood of aggregation. Additionally, when a higher concentration of T80 (1.2–1.5% *w*/*v*) was used in combination with a lower amount of mPEG-PCL, better stabilization occurred, resulting in particles with smaller sizes. For *h*ACP-PLHNs (Figure 5b), as the homogenization speed increases from 10,000 to 17,000 rpm, the energy applied is sufficient to reduce the PS, regardless of T80 concentration. Conversely, the effect of concentration of T80 was more pronounced at lower homogenization speeds (10,000 to 12,000 rpm). During this phase, the reduction in PS was primarily driven by a decrease in surface tension due to the presence of T80 rather than by the shear stress used to break up the globules or particles.

#### 3.3.2. Effect on EE (Y_2_)

**Figure 6:**
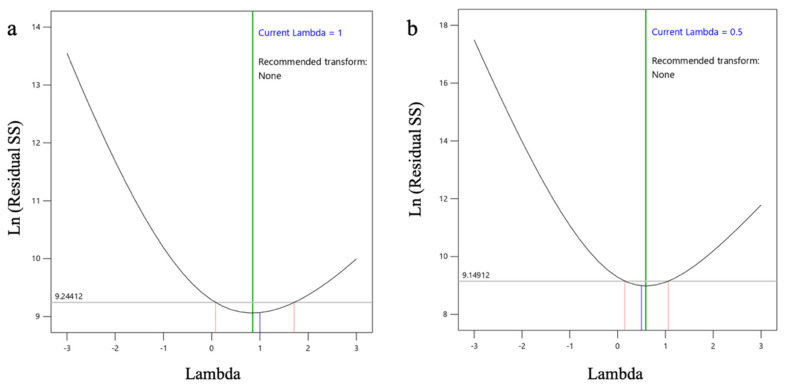
Box–Cox power plots showing the best transformation function for the EE (Y_2_) of *l*ACP-PLHNs (**a**) and *h*ACP-PLHNs (**b**).

The ANOVA (Table 5) suggests that EE of the *l*ACP-PLHNs required no transformation and was majorly influenced by homogenization speed (Factor C) and its interaction with the ratio of mPEG-PCL to lipid (interaction term AC). In contrast, the entrapment of ACP in *h*ACP-PLHNs was influenced by the ratio of mPEG-PCL to lipid. Both models were significant, and their goodness-of-fit can be remarked by the corresponding Box–Cox power transformation plots (Figure 6). The regression equations obtained for thee EE of *l*ACP-PLHNs and *h*ACP-PLHNs are presented in Equations (6) and (7), respectively.

(6) $$\mathrm{EE}\;\left(\%\right)=49.69+6.35\;A-7.92\;B+12.69\;C+8.78\;D-11.93\;\mathrm{AC}-7.26\;\mathrm{BC}$$

(7) $$\sqrt{\mathrm{EE}}\;\left(\%\right)=6.34+2.53\;A-0.2345\;B-0.0454\;C-0.1164\;D$$

**Figure 7:**
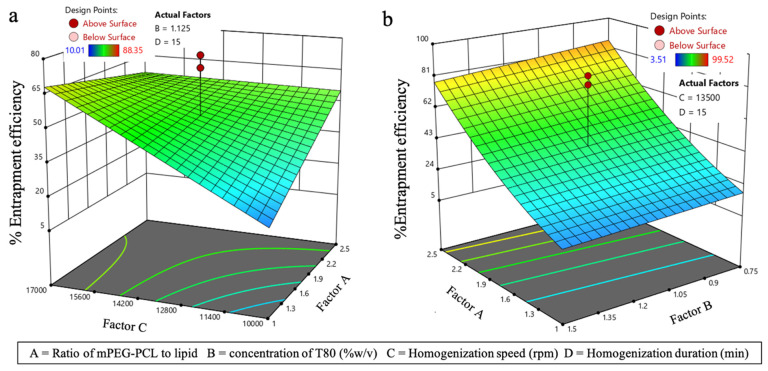
Three-dimensional response surface plots for EE (Y_2_) of *l*ACP-PLHNs (**a**) and *h*ACP-PLHNs (**b**).

In the 3D plot representing the EE of *l*ACP-PLHNs (Figure 7a), an overall increase in EE was observed when the homogenization speed (Factor C) was increased. At lower speeds, the formation of stable nanostructures can be less efficient, reducing the ability to entrap ACP. As previously mentioned, the EE of the nanocarriers depends on the separation process (in this case, centrifugation). If the nanostructures are not properly formed, they may rupture during centrifugation, causing ACP to be lost to the supernatant. At lower homogenization speeds, the energy applied could be insufficient for proper mixing and interaction between ACP and the excipients, resulting in an uneven drug distribution or lower entrapment. However, at speeds between 10,000 and 12,800 rpm, increasing the mPEG-PCL-to-lipid ratio (Factor A) improved EE. In polymer–lipid hybrid systems, the polymer phase plays a key role in trapping the drug and can act as a barrier to prevent premature drug release. A higher polymer content creates a larger matrix for drug encapsulation, especially when the homogenization speed is inadequate for achieving smaller particle sizes. Additionally, lower mPEG-PCL-to-lipid ratios lead to a relatively higher lipid content, resulting in a more fluid-like state prone to ACP leakage. At higher homogenization speeds (>13,000 rpm), increasing the mPEG-PCL-to-lipid ratio resulted in a moderate decrease in EE. The higher polymer content increases the rigidity of the formulation, making it more prone to breakage during homogenization. If the polymer matrix breaks, ACP previously entrapped within could be released, leading to a reduction in EE. However, for *h*ACP-PLHNs (Figure b), the mPEG-PCL-to-lipid ratio (Factor A) significantly impacted ACP entrapment. The mPEG-PCL-rich matrix enhances the structural integrity of the PLHNs and acts as a diffusion barrier, facilitating effective ACP entrapment. This improved structure also reduced the risk of particle rupture and ACP loss to the supernatant during centrifugation. Since ACP (>80 mg) was soluble in mPEG alone, the higher mPEG content in the co-polymer was expected to increase the affinity of ACP for the matrix, further enhancing entrapment. A slight increase in EE was observed when the concentration of T80 decreased from 1.5% to 0.75% *w*/*v*. This can be attributed to the fact that higher concentrations of T80 could enhance the solubilization of the available ACP, potentially altering its affinity for AP and thus slightly affecting EE.

### 3.4. Physical Characterization of ACP-PLHNs

**Figure 8:**
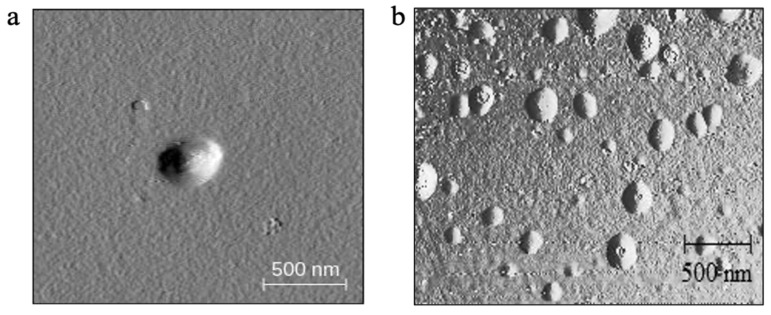
The amplitude forward scans of *l*ACP-PLLHNs (**a**) and *h*ACP-PLHNs (**b**) obtained by atomic force microscopy in AFM tapping mode.

The average PS of freshly prepared and reconstituted freeze-dried *l*ACP-PLHNs was 124.68 ± 8.87 nm, with a PDI of 0.380 ± 0.03, and 155.91 ± 10.32 nm, with a PDI of 0.361 ± 0.04, respectively. In contrast, the average PS of freshly prepared and reconstituted freeze-dried *h*ACP-PLHNs was 209.2 ± 13.30 nm, with a PDI of 0.357 ± 0.04, and 223.46 ± 12.11 nm, with a PDI of 0.382 ± 0.03. The structure of the resultant nanoparticles was anticipated to be mostly the matrix type (containing the homogenous matrix of co-polymer, lipid, and ACP in the core), with the mPEG chains tailed out in the hydrophilic environment, surrounded by a thin layer of surfactant covering the surface of the nanoparticles (as described in Figure 1). Hence, the increase in the average PS of *h*ACP-PLHNs may be attributed to the presence of larger or more hydrophilic components, such as the hydrophilic grade of mPEG-PCL, which could promote the formation of larger aggregates or a less compact nanoparticle structure. The average zeta potential (n = six batches each) for *l*ACP-PLHNs and *h*ACP-PLHNs was 4.58 mV and 3.22 mV, respectively. The images obtained from atomic force microscopy (Figure 8) reveal that both types of PLHNs exhibited spherical morphology with a smooth surface. An AFM was preferred over FE-SEM as PLHNs are often soft and non-conductive, and their morphology can be significantly altered by the high-energy electron beam in SEM. In contrast, AFM operates by scanning the surface with a fine tip, making it ideal for studying the native structure of these materials without causing deformation or evaporation, which could distort results in SEM.

### 3.5. Loading Efficiency and Entrapment Efficiency

The *l*ACP-PLHNs demonstrated an LE of 40.08 ± 3.43% and an EE of 79.41 ± 2.72%, while the *h*ACP-PLHNs showed a higher LE of 46.59 ± 1.33% and an EE of 85.33 ± 0.51%. The primary factors influencing both LE and EE are the solubility of the drug and its affinity for the matrix-forming excipients [32,33]. As previously discussed, since ACP is soluble in pure mPEG, a higher proportion of mPEG in the hydrophilic co-polymer (formed by mPEG to ε-caprolactone in the ratio of 4:9) allowed for better accommodation of ACP, resulting in a greater drug loading compared to the lipophilic grade (where the mPEG:ε-caprolactone ratio was 1:9).

### 3.6. Thermal Analysis

**Figure 9:**
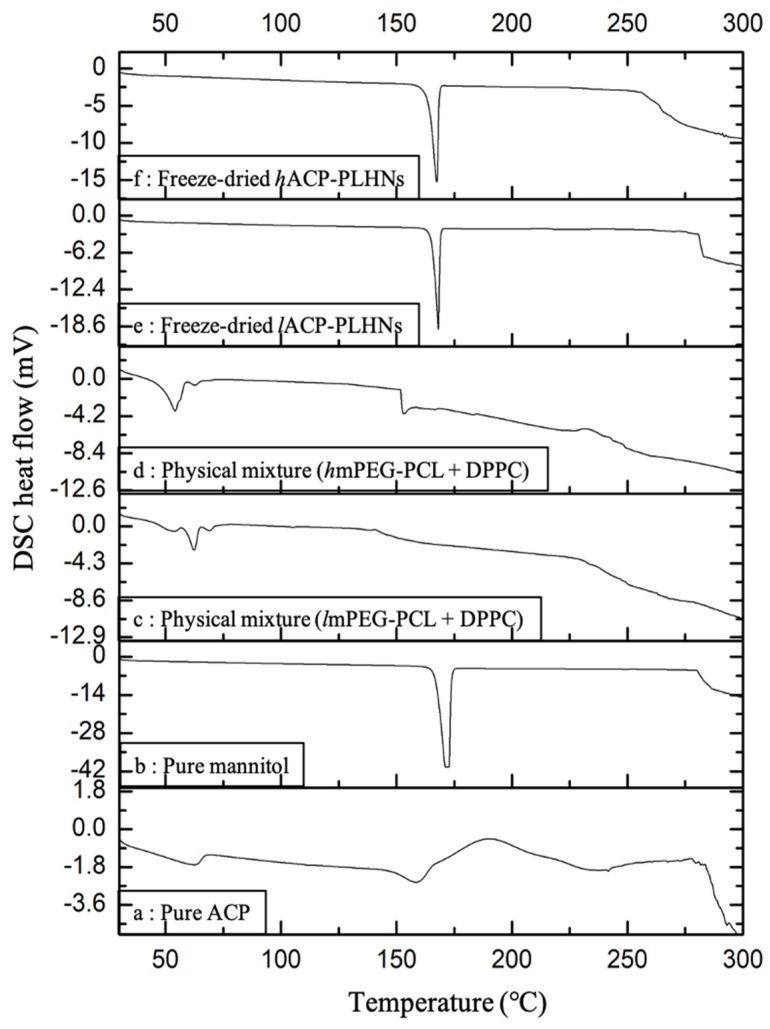
Overlay of DSC thermograms for pure ACP (**a**); pure mannitol (cryoprotectant) (**b**); physical mixture of *l*mPEG-PCL + DPPC (**c**); physical mixture of *h*mPEG-PCL + DPPC (**d**); freeze-dried *l*ACP-PLHNs (**e**); and freeze-dried *h*ACP-PLHNs (**f**). Endotherms and exotherms are represented by down and up, respectively.

Pure ACP, in the DSC overlay, exhibited two distinct endotherms at approximately 65 °C and 162 °C, followed by an exothermic peak around 198 °C (Figure 9a). The first endothermic peak around 65 °C could correspond to a less stable, disordered state of ACP, suggesting a transition to a more stable phase as heat is applied. The endothermic peak at 162 °C corresponds to the melting of the stable crystalline form, while the signals beyond 198 °C indicate the slow decomposition process of the sample. A similar DSC profile for an “S-type” polymorph of ACP has been reported by Matta et al., 2019 [34]. Mannitol, used as a cryoprotectant during freeze-drying, displayed a sharp melting endotherm at around 170 °C (Figure 9b). The physical mixture of both grades of co-polymers with DPPC showed an endothermic peak near 63 °C (Figure 9c,d). Both freeze-dried *l*ACP-PLHNs and *h*ACP-PLHNs exhibited a melting endotherm around 170 °C, corresponding to mannitol (Figure 9e and Figure 9f, respectively). The characteristic endothermic peaks of ACP were absent in both freeze-dried nanoparticle formulations, indicating the transformation of ACP into an amorphous form or its incorporation within the carrier at a molecular level. To further investigate these findings, p-XRD analysis of the samples was performed.

### 3.7. P-XRD Analysis

**Figure 10:**
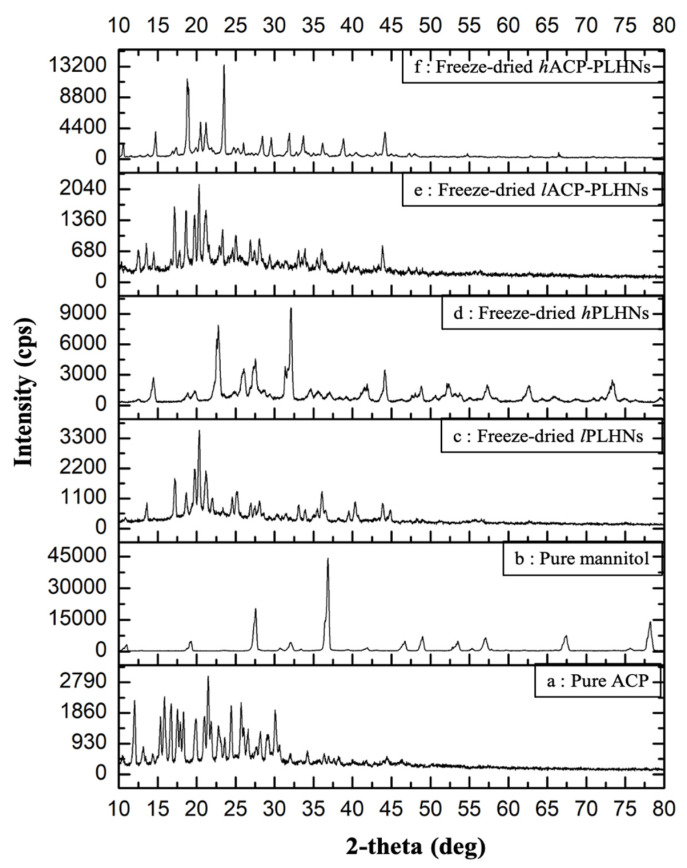
Overlay of X-ray diffractograms of pure ACP (**a**); pure mannitol (**b**); freeze-dried placebo *l*ACP-PLHNs (**c**) and *h*ACP-PLHNs (**d**); and freeze-dried *l*ACP-PLHNs (**e**) and *h*ACP-PLHNs (**f**).

The overlay of the diffraction patterns for various samples is shown in Figure 10. The diffraction pattern of pure ACP reveals intense characteristic peaks at 2-theta values of 10.5°, 12.1°, 15.8°, 24.4°, 25.7°, and 30.1° (Figure 10a). Pure mannitol, on the other hand, displays prominent peaks at 2-theta values of 27.5° and 36.3° (Figure 10b), along with several less intense peaks around 19° and 32° and between 45° and 80°. The freeze-dried placebo formulations prepared using the lipophilic grade of mPEG-PCL (*l*-PLHNs) exhibited characteristic peaks at 2-theta values of 13.6°, 17.2°, 19.8°, 20.3°, 24.6°, and 36.5° (Figure 10c), while those formulated using the hydrophilic grade of mPEG-PCL (*h*-PLHNs) showed peaks at 12.5°, 22.5°, 32°, and 44° (Figure 10d). Similarly, the diffractograms of both grades of ACP-PLHNs (Figure 10e,f) were almost identical, displaying similar peaks corresponding to those of their respective placebo nanoparticle formulations (Figure 10c,d). However, some peaks that were specifically present in pure ACP at 2-theta values of 10.5°, 25.7°, and 30.1° were missing in both ACP-PLHNs. This supports the observations made in the DSC analysis that ACP is present either in an amorphous or molecularly dispersed form in both nanoparticles.

### 3.8. Estimation of Residual Solvents

According to the ICH guideline for residual solvents (ICH Q3C(R8)), CHCl_3_ is classified as a Class II solvent, with a permissible daily exposure (PDE) limit of 60 ppm/day (or 400 µL/day) [35]. This limit is supported by a toxicological report from the Agency for Toxic Substances and Disease Registry, Public Health Service and the U.S. Department of Health and Human Services, which highlights the potential respiratory, hepatic, renal, neurological, and developmental risks associated with CHCl_3_ exposure [36]. As shown in Figure S1a, the CHCl_3_ content in freeze-dried *l*ACP-PLHNs and *h*ACP-PLHNs was well below the PDE limit but also significantly lower than the method’s lower limit of quantification (1.5 ppm or 10 µL/mL) (Figure S1b). The analysis confirms that the CHCl_3_ levels in the final product are far beneath established safety thresholds, ensuring compliance with regulatory standards and minimizing potential health risks.

### 3.9. In Vitro Dissolution Study

The aqueous solubility of ACP decreases as the pH of the medium increases—29.2 mg/mL at pH 1 to 0.159 mg/mL at pH 4.5 to 0.0523 mg/mL at pH 6.8 [37]. The use of SDS in in vitro dissolution studies of ACP was previously reported by Pepin and colleagues. To maintain sink conditions, 0.5% *w*/*v* SDS was added to the dissolution medium with a pH ≥ 4.5 due to the low solubility of the drug at those pH conditions. Additionally, the volume of the dissolution medium for each pH condition was set at three times the minimum volume required to dissolve the entire drug present in the nanoparticle formulation in the dissolution medium while mimicking the physiological fluid volumes present at the relevant absorption sites.

**Figure 11:**
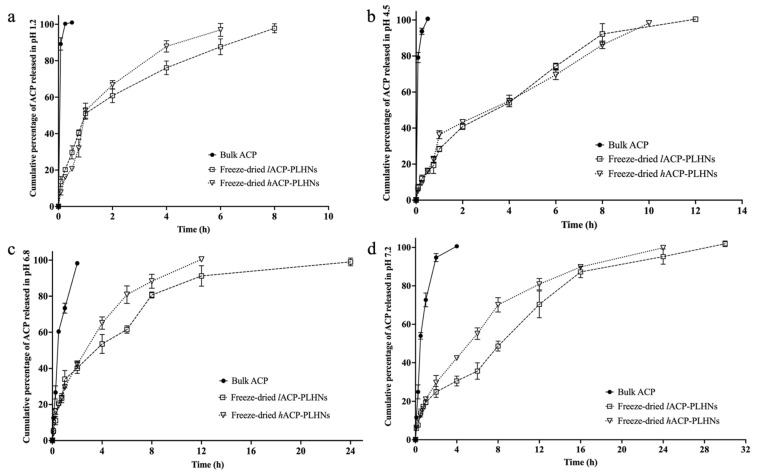
Comparative in vitro dissolution between bulk ACP, freeze-dried *l*ACP-PLHNs and freeze-dried *h*ACP-PLHNs at pH 1.2 (**a**); 4.5 (**b**); 6.8 (**c**); and 7.2 (**d**) under sink conditions.

Under sink conditions, the bulk ACP was completely dissolved within 30 min in dissolution media with pH 1.2 and 4.5 (Figure 11a and Figure 11b, respectively) and within 2 h and 4 h in dissolution media with pH 6.8 and 7.2, respectively (Figure 11c and Figure 11d, respectively). In the case of both types of ACP-PLHNs, the release was sustained and primarily dependent on the types of lipids and polymers used, as well as the affinity of ACP for the hybrid matrix. The drug release from both ACP-PLHNs could be due to the diffusion of ACP through the carrier matrix and/or the erosion of the carrier matrix, resulting in the release of ACP. The outer surface of the nanoparticles is exposed to the aqueous environment, making the mPEG-PCL co-polymer susceptible to degradation through hydrolysis of the ester bonds between mPEG and PCL [38]. This degradation creates pores and cavities in the nanoparticle structure, facilitating the diffusion and release of the entrapped ACP into the dissolution medium. Additionally, both DPPC and lecithin (amphiphilic in nature) interact with ACP due to its affinity for these lipids, forming pockets or complexes that not only enhance the drug’s dispersion in the medium but also maintain it in a solubilized state, preventing crystallization and precipitation. At higher pH conditions (pH 6.8 and 7.2), over 80% of ACP was released from the *h*ACP-PLHNs within 8 to 12 h. Complete release (100%) occurred within 6, 10, 12, and 24 h in dissolution media with pH 1.2, 4.5, 6.8, and 7.2, respectively. In contrast, ACP release from *l*ACP-PLHNs was slower, extending over 8, 12, 24, and 30 h in dissolution media with pH 1.2, 4.5, 6.8, and 7.2, respectively. These differences in release profiles could be due to the differences in the polymer molecular weight and block length between the two formulations. The increased mPEG content in the co-polymer used to formulate *h*ACP-PLHNs enhances the water affinity of PLHNs, accelerating the rate of hydrolytic degradation, with faster pore formation and a more open matrix, allowing quicker diffusion of ACP. Furthermore, the solubilizing effect of mPEG (due to its surfactant-like behavior, owing to its inherent hydrophilicity) may enhance the matrix hydration, reducing the viscosity of the nanoparticle matrix and finally accelerating the diffusion of ACP out of the matrix. On the other hand, *l*ACP-PLHNs, which were formulated using a more hydrophobic co-polymer (with a lower proportion of mPEG and longer PCL blocks), exhibit slower drug release due to several interconnected factors. The reduced hydrophilicity of the polymer matrix limits water uptake and penetration, which, in turn, slows down the hydrolytic degradation of the ester bonds in the PCL backbone. Moreover, the higher molecular weight of the PCL segment contributes to enhanced polymer stability and reduced degradation kinetics, as longer polymer chains require more extensive hydrolysis to break down into soluble oligomers.

Both types of PLHNs exhibited release profiles that closely followed the Korsmeyer–Peppas model, with R^2^ values > 0.98 for *l*ACP-PLHNs and >0.97 for *h*ACP-PLHNs. The value of n (the release exponent) for *l*ACP-PLHNs ranged from 0.441 to 0.560, indicating that the release of ACP was primarily driven by drug diffusion through the carrier matrix. In contrast, for *h*ACP-PLHNs, the n value ranged from 0.547 to 0.619, suggesting that the release was governed by a combination of drug diffusion and polymeric chain relaxation.

### 3.10. Stability Studies

**Figure 12:**
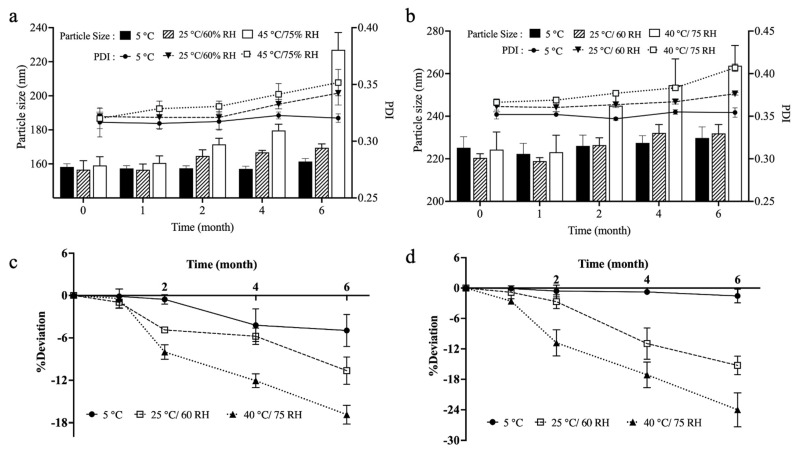
Stability analysis over 6 months, illustrating changes in PS and PDI of *l*ACP-PLHNs (**a**) and *h*ACP-PLHNs (**b**) and variations in EE of *l*ACP-PLHNs (**c**) and *h*ACP-PLHNs (**d**) under different storage conditions.

The freeze-dried samples of both types of ACP-PLHNs subjected to stability studies were analyzed based on three critical aspects: PS, PDI, and EE. Figure S2a,b show the physical appearance of *l*ACP-PLHNs and *h*ACP-PLHNs, respectively, under various storage conditions, both immediately after preparation and after six months of storage. For both *l*ACP-PLHNs and *h*ACP-PLHNs, a noticeable change in color was observed. Temperature plays a significant role in the stability of these formulations, particularly with lipids like DPPC and lecithin, whose phase transition temperatures range from 40 to 42 °C [39]. At elevated temperatures (e.g., 45 °C), the peroxidation of fatty acid chains in DPPC and lecithin may contribute to the darkening of the formulations. The DSC thermograms of physical mixtures of both types of mPEG-PCL individually with DPPC exhibit a lower melting temperature (Section 3.6, Figure 9c,d). Additionally, mPEG, being more hydrophilic than the PCL counterpart of the co-polymer, may accelerate degradation by facilitating the uptake of water molecules under higher humidity conditions (60–75% RH). This is reflected in a more pronounced color change in *h*ACP-PLHNs at 45 ± 2 °C/75 ± 5% RH compared to *l*ACP-PLHNs. Conversely, samples at 25 ± 2 °C/60 ± 5% RH exhibit more gradual changes, while those stored at 5 ± 2 °C show minimal alterations, maintaining their physical integrity and preventing rehydration and phase transitions. These observations were supported by the change in the PS and PDI of the formulations under various conditions. For *l*ACP-PLHNs (Figure 12a), a 1.2-fold (*p* < 0.05) and 1.43-fold (*p* < 0.001) increase in PS was observed when stored at 25 ± 2 °C/60 ± 5% RH and 45 ± 2 °C/75 ± 5% RH, respectively. The PDI of *l*ACP-PLHNs increased from 0.250 to 0.358 at 45 ± 2 °C/75 ± 5% RH. For *h*ACP-PLHNs, an increase of 18% (*p* < 0.001) in PS and 11.10% (*p* < 0.001) in PDI was observed at 45 ± 2 °C/75 ± 5% RH (Figure 12b). The EE of ACP in *l*ACP-PLHNs deviated up to −9.34% at 25 ± 2 °C, while a deviation of −21.20% at 45 ± 2 °C/75 ± 5% RH was observed (Figure 12c). Similar observations were also reported in the case of *h*ACP-PLHNs, where the EE deviated by −15.25% and −23.99% by the end of the storage period when stored at 25 ± 2 °C/60 ± 5% RH and 45 ± 2 °C/75 ± 5% RH, respectively (Figure 12d). At higher temperature and humidity conditions, the phase transitions; degradation of both polymer and lipid components; and an uptake of water disrupted the structure of the ACP-PLHNs, resulting in the release of the encapsulated ACP. Also, as the moisture content increases with high humidity, the formulation becomes more susceptible to rehydration and aggregation, both of which lead to a decrease in the EE of nanoparticles.

### 3.11. Preventive Evaluations

**Figure 13:**
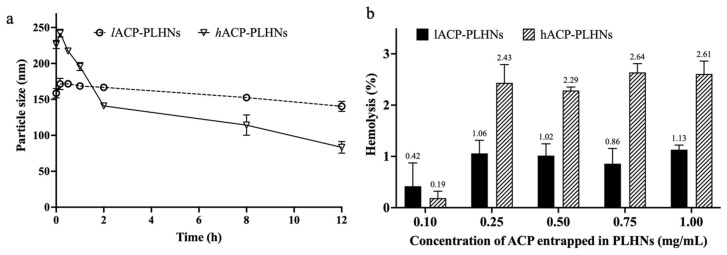
Graphical representation of colloidal stability of *l*ACP-PLHNs and *h*ACP-PLHNs in pH 7.2 buffer (**a**) and their hemolytic assay conducted by UV-visible spectrometry (**b**).

**Figure 14:**
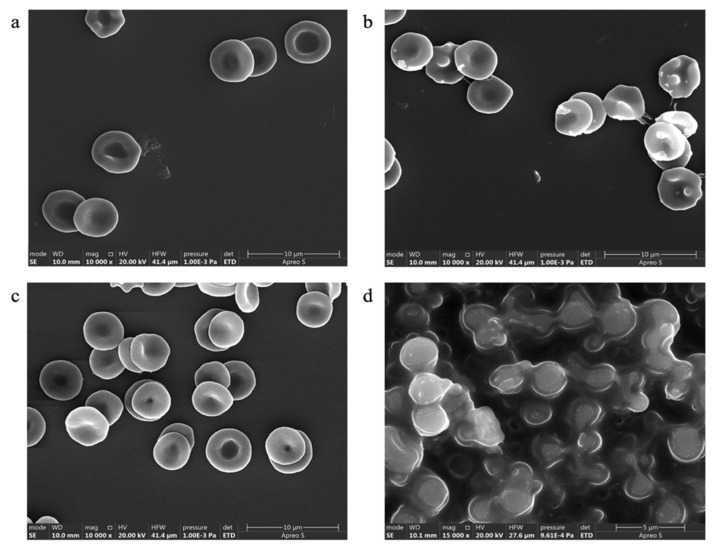
Comparative morphological analysis of RBCs in presence of *l*ACP-PLHNs (**a**); *h*ACP-PLHNs (**b**); negative control (**c**); and positive control (**d**).

The PS of nanoparticles was identified as one of the critical responses during the optimization of both types of ACP-PLHNs. As a result, concerns arose regarding potential particle aggregation or component precipitation in the systemic circulation following the direct uptake of the nanoparticles from the gastrointestinal tract upon their oral administration. The presence of various ions or plasma proteins could exacerbate these issues, potentially leading to vascular blockages and severe adverse effects. To mitigate these risks, the in vitro colloidal stability of the ACP-PLHNs was rigorously evaluated under conditions simulating blood or plasma (a pH 7.2 buffer) [40]. DLS analysis of samples reveals an 11.55% and a 63.20% decrease in PS of *l*ACP-PLHNs and *h*ACP-PLHNs over 24 h, respectively, which effectively rules out aggregation (Figure 13a). An increase in the rate of decrease of PS for *h*ACP-PLHNs may be attributed to the affinity of the system towards aqueous medium and faster degradation of the carrier system, as detailed in Section 3.9. Furthermore, a hemolysis assay, conducted using a UV-visible spectrophotometer, confirmed the safety of the formulation, with a hemolysis rate of <1.2% for *l*ACP-PLHNs and <2.65% for *h*ACP-PLHNs when RBCs were incubated with PLHNs containing the maximum ACP concentration (1 mg/mL) (Figure 13b). The FE-SEM analysis of the RBCs collected at 0.75 h (Figure 14) after administration of both types of ACP-PLHNs illustrates no morphological change (Figure 14a,b) when compared to the negative control (Figure 14c). Hence, the prepared formulations at a dose of 30 mg/kg did not show any toxicity and were considered safe for conducting preclinical in vivo PK studies.

### 3.12. In Vivo PK and Tissue Distribution Studies

**Figure 15:**
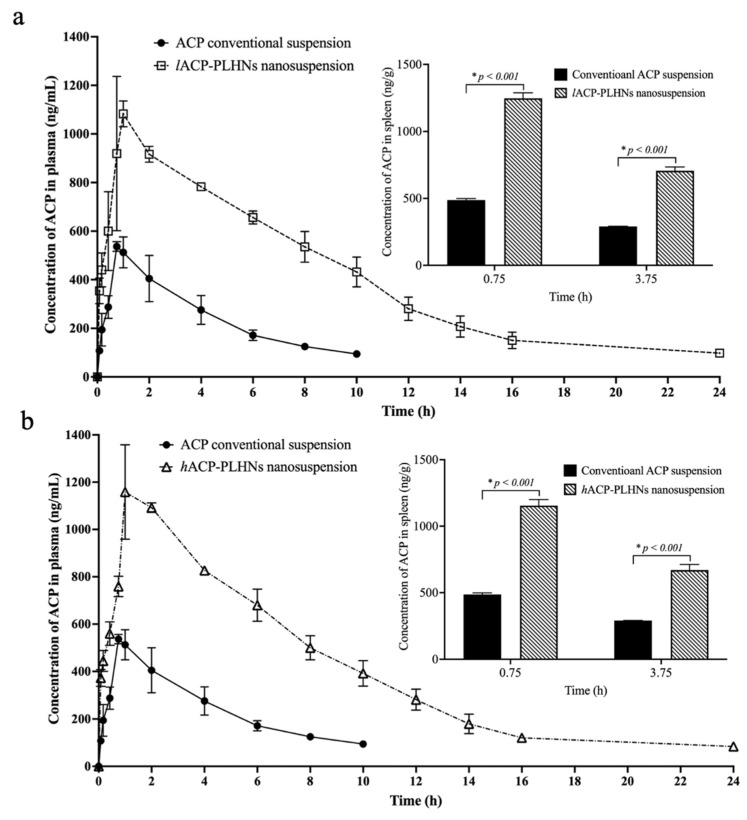
Comparative plasma concentrations after administration of conventional ACP suspension with *l*ACP-PLHN (**a**) and *h*ACP-PLHN (**b**) nanosuspensions. The plasma profile of conventional ACP suspension was adopted from Sinha et al., 2024 [18]. Inset plots represent comparison between concentration of ACP in spleen after the oral administration of conventional ACP suspension (adopted from [29]) and *l*ACP-PLHN (**a**) and *h*ACP-PLHN (**b**) nanosuspensions.

The oral administration of both *l*ACP-PLHNs and *h*ACP-PLHNs resulted in a more than 2-fold (*p* < 0.001) improvement in the plasma exposure (expressed in terms of AUC_0-tlast_, Table 6) (Figure 15a and Figure 15b, respectively) compared to the conventional suspension of the drug. Both nanosuspensions resulted in a comparable increase in the absolute oral bioavailability (> 80%) of ACP (Table 6). The PLHNs, by virtue of their nanometric size and amphiphilic nature, modified the pH-dependent solubility of ACP and improved the apparent solubility of ACP in the intestinal fluids at a pH > 6.8. The nanoparticle formulations enhanced the dissolution rate of the drug and improved the absorption of the drug specifically from the intestinal fluids (Section 3.9, Figure 11c). Also, the biocompatibility of the delivery system, along with a PS in the range of 150–250 nm, could have favored the direct uptake of the nanoparticles into the systemic circulation [33]. Further, as ACP is entrapped in a carrier system, it is not exposed to the gut wall metabolizing enzymes. However, the concentration levels at T_max_ were higher for the *h*ACP-PLHNs than the *l*ACP-PLHNs. This may be attributed to the faster dissolving behavior of the former due to the presence of more amounts of mPEG than the latter one. Further, due to the slow degradation property of the PCL component of the mPEG-PCL block co-polymer, a prolonged mean residence time (> 6 h) was observed for the ACP-PLHN nanosuspension compared to the conventional suspension (~3.65 h).

In patients suffering from advanced stages of CLL, splenomegaly is frequently observed due to the uncontrolled proliferation of malignant B cells [41,42,43]. Hence, to effectively treat the malignant B-cell receptor signaling axis within the tumor microenvironment, it is very important to achieve higher concentrations of ACP in the spleen. Following oral administration, both ACP-PLHNs achieved more than 2-fold higher (*p* < 0.001) concentrations of the drug in spleens at 0.75 h and 3.75 h compared to the conventional suspension of ACP (the values for the conventional ACP suspension were adopted from previously published data by the same research group [29]) (inset plots in Figure 15a,b). The higher ACP levels in the spleen could help mitigate this condition by targeting and inhibiting the growth of malignant cells in the tissue.

## 4. Conclusions

Two grades of mPEG-PCL co-polymers, one lipophilic and one hydrophilic, were synthesized via ring-opening polymerization, yielding molecular weights of 9817.67 Da and 23,615.84 Da, respectively. These co-polymers were then used to formulate two distinct types of PLHNs in combination with DPPC and lecithin, which were optimized using a cCCD. The *l*ACP-PLHNs exhibited a PS of 155.91 nm and a drug loading of 40.08%, while the *h*ACP-PLHNs demonstrated a larger PS of 223.46 nm and a higher drug loading of 46.59%. Notably, the *h*ACP-PLHNs exhibited a higher in vitro dissolution rate, emphasizing that a higher mPEG proportion can enhance the release rate of hydrophobic drugs like ACP and maintain its solubility. Stability studies indicate that *l*ACP-PLHNs were more stable than *h*ACP-PLHNs, likely due to fewer molecular rearrangements in the lipophilic formulation. Both formulations significantly increased the oral bioavailability of ACP, with an approximate 3.9-fold (*p* < 0.001) improvement compared to conventional ACP.

In summary, PLHNs containing mPEG-PCL co-polymers offer a promising approach to improving the oral bioavailability of ACP, with formulation characteristics, including polymer composition, playing a key role in optimizing dissolution behavior, stability, and pharmacokinetics. These findings underscore the potential of mPEG-PCL-based PLHNs as a viable strategy for enhancing the therapeutic efficacy of hydrophobic drugs.

## Figures and Tables

**Table 1:** The critical factors, levels (at their original or nominal scale), and response variables used in the cCCD.

Factor Code	Factors and Units	High Level (+1)	Low Level (−1)	Alpha High (+α)	Alpha Low (−α)	Center Point Level
A	Ratio of mPEG-PCL to lipid	2.5	1	3.25	0.25	1.75
B	Concentration of T80 (% *w*/*v*)	1.5	0.75	1.875	0.375	1.125
C	Homogenization speed (rpm)	17,000	10,000	18,800	3800	11,300
D	Homogenization duration (min)	20	10	25	5	15
The critical responses of both *l*ACP-PLHNs and *h*ACP-PLHNs.						
Y_1_	PS (nm)		Less than 250 nm is desirable			
Y_2_	PDI		Less than 0.6 is desirable			
Y_3_	EE		Higher than 50% is desirable			

**Table 2:** The preliminary batches of *l*ACP-PLHNs and *h*ACP-PLHNs to determine the critical material attributes and critical process parameters and their levels affecting the CRVs.

Batch No.	Material Attributes		Process Parameters of High Shear Homogenization	CRVs		
	Ratio of mPEG-PCL to Lipid	Concentration of T80 (% *w*/*v*)		PS (nm)	PDI	EE (%)
*Batches prepared using lipophilic grade of mPEG-PCL*						
01-L	2:1	0.25	Speed = 10,000 rpm Duration = 20 min	220.38	0.402	64.96
02-L	1:1	0.5	Speed = 10,000 rpm Duration = 15 min	218.64	0.323	5.82
03-L	2:1	1.0	Speed = 15,000 rpm Duration = 10 min	153.81	0.226	56.17
04-L	2:1	1.0	Speed = 20,000 rpm Duration = 15 min	210.55	0.372	43.60
*Batches prepared using hydrophilic grade of mPEG-PCL*						
01-H	2:1	0.25	Speed = 10,000 rpm Duration = 20 min	280.19	0.439	80.84
02-H	1:1	0.5	Speed = 10,000 rpm Duration = 15 min	312.24	0.401	38.47
03-H	2:1	1.0	Speed = 15,000 rpm Duration = 10 min	204.75	0.211	62.32
04-H	2:1	0.75	Speed = 20,000 rpm Duration = 15 min	134.17	0.238	60.42

Note: CRVs = critical response variables; PS = particle size; PDI = polydispersity index; EE = entrapment efficiency. DPPC was used as the lipid; the temperature of AP was maintained at 55 °C; and the rate of addition of OP to AP was maintained at 0.5 mL/min across all batches.

**Table 3:** The experimental runs, as obtained from the cCCD by Design Expert software, for optimizing *l*ACP-PLHNs, along with the responses observed for each run.

Std Run	Factor A	Factor B	Factor C	Factor D	PS (nm)	EE (%)
9	1	0.75	10,000	20	147.8	10.01
10	2.5	0.75	10,000	20	114.8	70.4
21	1.75	1.125	6500	15	175.8	13.56
7	1	1.5	17,000	10	74.78	27.73
26	1.75	1.125	13,500	15	100.3	71.17
22	1.75	1.125	20,500	15	185.3	84.57
20	1.75	1.875	13,500	15	143.4	25.36
27	1.75	1.125	13,500	15	135.9	32.25
5	1	0.75	17,000	10	106.8	85.46
19	1.75	0.375	13,500	15	174.4	19.92
28	1.75	1.125	13,500	15	190.1	31.83
16	2.5	1.5	17,000	20	127.9	52.54
14	2.5	0.75	17,000	20	106.6	86.27
13	1	0.75	17,000	20	160.1	88.35
3	1	1.5	10,000	10	172.9	12.46
2	2.5	0.75	10,000	10	162.8	58.97
15	1	1.5	17,000	20	114.2	77.91
25	1.75	1.125	13,500	15	117.4	76.8
23	1.75	1.125	13,500	5	165.5	46.85
17	0.25	1.125	13,500	15	96.18	14.83
12	2.5	1.5	10,000	20	215.3	71.84
18	3.25	1.125	13,500	15	167.2	55.15
11	1	1.5	10,000	20	132.6	30.1
24	1.75	1.125	13,500	25	137.1	82.88
4	2.5	1.5	10,000	10	149.7	32.8
1	1	0.75	10,000	10	165.1	50.18
6	2.5	0.75	17,000	10	121.4	68.84
8	2.5	1.5	17,000	10	154.4	12.23

Note: The standard run number defines a standard label to describe the geometric location of the run in the space. Factor A = the ratio of mPEG-PCL (lipophilic grade) to lipid; B = the concentration of T80 (% *w*/*v*); C = homogenization speed (rpm); and D = homogenization duration (min). The temperature of AP was maintained at 55 °C; the ratio of OP to AP was maintained at 1:25; and the rate of the addition of OP to AP was maintained at 0.5 mL/min across all the trials. The responses are reported as the mean of three independent measurements.

**Table 4:** The experimental runs, as obtained from the cCCD by Design Expert software, for optimizing *h*ACP-PLHNs, along with the responses observed for each run.

Std Run	Factor A	Factor B	Factor C	Factor D	PS (nm)	%EE
13	1	0.75	17,000	20	304.9	15.5
7	1	1.5	17,000	10	158.8	7.51
12	2.5	1.5	10,000	20	130.9	63.2
14	2.5	0.75	17,000	20	163.2	81.63
9	1	0.75	10,000	20	186.8	7.68
15	1	1.5	17,000	20	169.6	6.43
19	1.75	0.375	13,500	15	165.4	71.08
24	1.75	1.125	13,500	25	309.5	61.66
28	1.75	1.125	13,500	15	186.9	79.27
26	1.75	1.125	13,500	15	212.3	25.24
18	3.25	1.125	13,500	15	188.2	77.3
5	1	0.75	17,000	10	135.1	6.57
16	2.5	1.5	17,000	20	97.37	57.49
6	2.5	0.75	17,000	10	112.5	93.67
27	1.75	1.125	13,500	15	226.9	5.75
23	1.75	1.125	13,500	5	261.9	44.15
1	1	0.75	10,000	10	159.7	8.7
20	1.75	1.875	13,500	15	128.4	50.42
4	2.5	1.5	10,000	10	114	92.95
21	1.75	1.125	6500	15	198.8	59.51
22	1.75	1.125	20,500	15	50.11	48.37
17	0.25	1.125	13,500	15	171.7	8.07
10	2.5	0.75	10,000	20	243.3	91.07
3	1	1.5	10,000	10	133.8	16.4
2	2.5	0.75	10,000	10	142.3	78.14
25	1.75	1.125	13,500	15	202.1	84.61
8	2.5	1.5	17,000	10	107.9	99.52
11	1	1.5	10,000	20	130.3	3.51

Note: The standard run number defines a standard label to describe the geometric location of the run in the space. Factor A = the ratio of mPEG-PCL (hydrophilic grade) to lipid; B = the concentration of T80 (% *w*/*v*); C = homogenization speed (rpm); and D = homogenization duration (min). The temperature of AP was maintained at 55 °C; the ratio of OP to AP was maintained at 1:25; and the rate of the addition of OP to AP was maintained at 0.5 mL/min across all the trials. The responses are reported as the mean of three independent measurements.

**Table 5:** ANOVA for PS (Y_1_) and EE (Y_2_) for *l*ACP-PLHNs and *h*ACP-PLHNs.

Source	Y_1_ = PS		Y_2_ = EE	
	*l*ACP-PLHNs	*h*ACP-PLHNs	*l*ACP-PLHNs	*h*ACP-PLHNs
**Transformation**	Power	Inverse square root	None	Square root
**Model**	0.0496	<0.0001	0.0041	<0.0001
**A**	0.0966	0.6344	0.1409	<0.0001
**B**	0.7748	0.0004	0.0701	0.4997
**C**	0.0572	<0.0001	0.006	0.8956
**D**	-	0.0043	0.0463	0.7366
**AB**	0.0542	0.2561	-	-
**AC**	-	0.0042	0.0288	-
**BC**	-	-	0.1677	-
**BD**	-	0.0042	-	-
**A^2^**	-	0.1406	-	-
**B^2^**	-	0.0027	-	-
**C^2^**	-	<0.0001	-	-
**D^2^**	-	0.0452	-	-
**A^2^C**	-	<0.0001	-	-
**AB^2^**	-	0.0243	-	-
**Lack of Fit**	0.7541	0.1593	0.7626	0.9971

Note: *p*-values < 0.05 denote significant model terms. However, the insignificant terms had to be included in the model to maintain the significance or model hierarchy. Factor A = the ratio of mPEG-PCL to lipid; B = the concentration of T80 (% *w*/*v*); C = homogenization speed (rpm); and D = homogenization duration (min). The symbol “-” against a term indicates that term is not included in the model.

**Table 6:** Pharmacokinetic parameters obtained after oral administration of conventional ACP suspension and *l*ACP-PLHN and *h*ACP-PLHN nanosuspensions.

Parameters (Units)		Conventional ACP Suspension ^^^	*l*ACP-PLHN Nanosuspension	*h*ACP-PLHN Nanosuspension
$C_{\max}$	ng/mL	558.25 ± 22.45	1140.94 ± 133.72	1207.35 ± 116.64
$T_{\max}$	h	0.75	1	1
${\mathrm{AUC}}_{0-\mathrm{tlast}}$	h * ng/mL	2447.85 ± 269.42	9530.43 ± 634.51	9510.17 ± 281.71
F_abs_ ^#^	-	26.83 ± 1.35	83.69 ± 5.57	83.51 ± 2.48
F_rel_ *	-	-	3.96 ± 0.73	3.93 ± 0.49
${\mathrm{MRT}}_{0-\mathrm{tlast}}$	h	3.65 ± 0.15	7.23 ± 0.40	6.76 ± 0.22

Note: All values are reported as mean ± SD, T_max_ is reported as median (n = 3 independent determinations). ^^^ Data of conventional ACP suspension is reproduced from Sinha et al., 2024 [18]. ^#^ Absolute bioavailability of conventional ACP suspension and *l*ACP-PLHN and *h*ACP-PLHN nanosuspensions were determined using $F_{\mathrm{abs}}=\left[\frac{{\left({\mathrm{AUC}}_{0-\mathrm{tlast}}\right)}_{\mathrm{Oral}}}{{\left({\mathrm{AUC}}_{0-\mathrm{tlast}}\right)}_{\mathrm{IV}}}\times \frac{{\mathrm{Dose}}_{\mathrm{IV}}}{{\mathrm{Dose}}_{\mathrm{Oral}}}\right]\times 100$. * Relative bioavailability of *l*ACP-PLHN and *h*ACP-PLHN nanosuspensions were determined using $F_{\mathrm{rel}}=\left[\frac{{\left({\mathrm{AUC}}_{0-\mathrm{tlast}}\right)}_{l\mathrm{ACP}-\mathrm{PLHNs}\;\mathrm{or}\;h\mathrm{ACP}-\mathrm{PLHNs}\;\mathrm{nanosuspension}}}{{\left({\mathrm{AUC}}_{0-\mathrm{tlast}}\right)}_{\mathrm{Conventional}\;\mathrm{ACP}\;\mathrm{suspension}}}\right]$.

## Data Availability

The original dataset/output obtained from the study is included in the article main text. However, any other datasets generated during and/or analyzed during the current study will be available from the corresponding author, Punna Rao Ravi (rpunnarao@hyderabad.bits-pilani.ac.in) upon reasonable request.

## Supplementary Materials

The following supporting information can be downloaded at: https://www.mdpi.com/article/10.3390/pharmaceutics17060774/s1, Figure S1: Overlay of the chromatograms between the calibration standards and *l*ACP-PLHNs and *h*ACP-PLHNs (a) along with a comparative analysis between lowest calibration standard (10 μL/mL), *l*ACP-PLHNs and *h*ACP-PLHNs (solubilized freeze-dried ACP-PLHNs), and blank DMSO (b). Figure S2: Stability analysis over 6 months, illustrating changes in physical appearance of *l*ACP-PLHNs (a) and *h*ACP-PLHNs (b) under different storage conditions.
