# Supplementary material for: mPEG-PCL Nanoparticles to Improve Oral Bioavailability of Acalabrutinib: Effect of Polymer Lipophilicity and Hydrophilicity on Physicochemical Properties and In Vivo Performance in Rats
Source: Pharmaceutics. 2025 Jun 13;17(6):774. doi: 10.3390/pharmaceutics17060774 (PMC12197210; doi:10.3390/pharmaceutics17060774)
Supplement: Supplementary file 1 [file pharmaceutics-17-00774-s001.zip › pharmaceutics-3656483-supplementary.pdf]

mPEG-PCL Nanoparticles to Improve Oral Bioavailability of Acalabrutinib: Effect of Polymer Lipophilicity and Hydrophilicity on Physicochemical Properties and In Vivo Performance in Rats

Swagata Sinha [1], Punna Rao Ravi [1,*], Sahadevan Rajesh Rashmi [1] and Łukasz Szeleszczuk [2]

[1] Department of Pharmacy, Birla Institute of Technology and Science, Pilani, Hyderabad Campus, Jawahar Nagar, Kapra Mandal, Medchal District, Telangana 500078, India; p20210055@hyderabad.bits-pilani.ac.in (S.S.); p20220224@hyderabad.bits-pilani.ac.in (S.R.R.)
[2] Department of Organic and Physical Chemistry, Faculty of Pharmacy, Medical University of Warsaw, 1 Banacha Str., 02-093 Warsaw, Poland; lszeleszczuk@wum.edu.pl
* Correspondence: rpunnarao@hyderabad.bits-pilani.ac.in

Supplementary Data

- **Estimation of residual solvents**

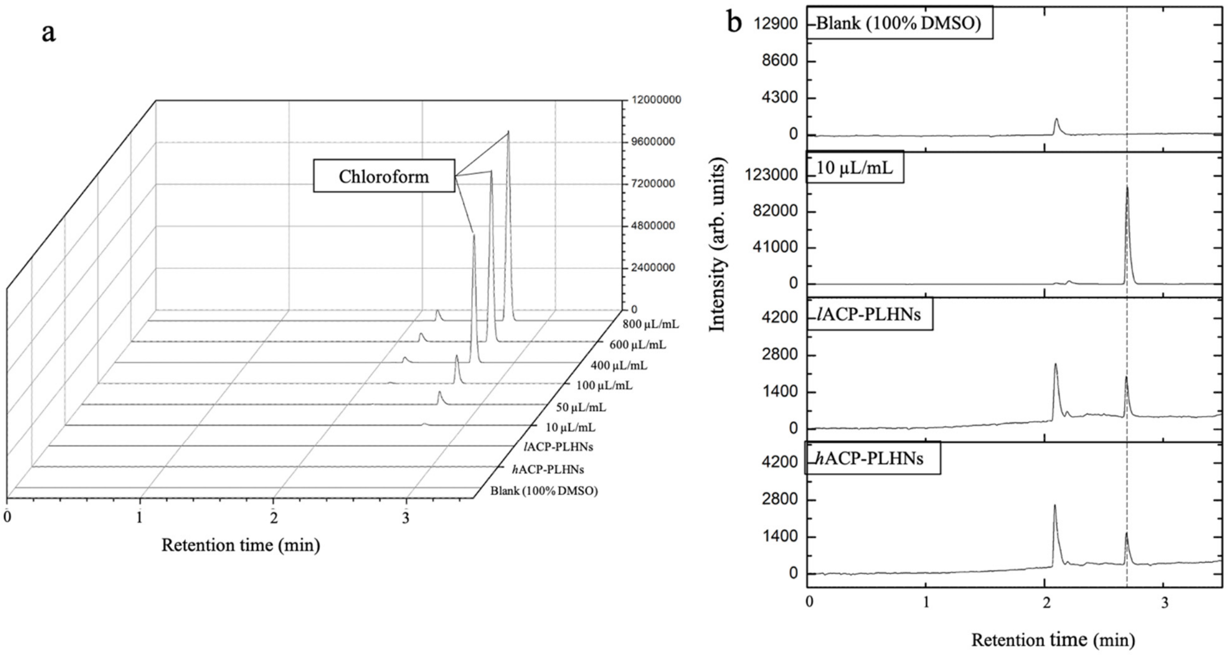

Figure S1 : Overlay of the chromatograms between the calibration standards and *l*ACP-PLHNs and *h*ACP-PLHNs (a) along with a comparative analysis between lowest calibration standard (10 µL/mL), *l*ACP-PLHNs and *h*ACP-PLHNs (solubilized freeze-dried ACP-PLHNs), and blank DMSO (b).

- **Stability studies**

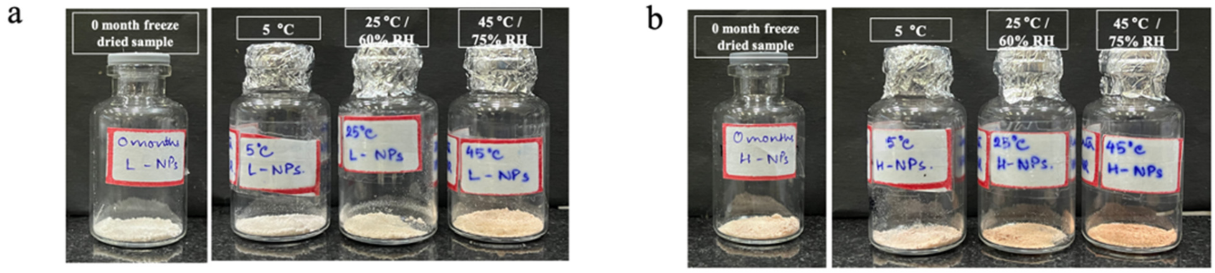

Figure S2 : Stability analysis over 6 months, illustrating changes in physical appearance of *l*ACP-PLHNs (a) and *h*ACP-PLHNs (b) under different storage conditions.
